# Development of a novel score for the prediction of hospital mortality in patients with severe sepsis: the use of electronic healthcare records with LASSO regression

**DOI:** 10.18632/oncotarget.17870

**Published:** 2017-05-15

**Authors:** Zhongheng Zhang, Yucai Hong

**Affiliations:** ^1^ Department of Emergency Medicine, Sir Run-Run Shaw Hospital, Zhejiang University School of Medicine, Hangzhou, 310016, China

**Keywords:** sepsis, LASSO, prediction

## Abstract

**Background and objective:**

There are several disease severity scores being used for the prediction of mortality in critically ill patients. However, none of them was developed and validated specifically for patients with severe sepsis. The present study aimed to develop a novel prediction score for severe sepsis.

**Results:**

A total of 3206 patients with severe sepsis were enrolled, including 1054 non-survivors and 2152 survivors. The LASSO score showed the best discrimination (area under curve: 0.772; 95% confidence interval: 0.735-0.810) in the validation cohort as compared with other scores such as simplified acute physiology score II, acute physiological score III, Logistic organ dysfunction system, sequential organ failure assessment score, and Oxford Acute Severity of Illness Score. The calibration slope was 0.889 and Brier value was 0.173.

**Materials and Methods:**

The study employed a single center database called Medical Information Mart for Intensive Care-III) MIMIC-III for analysis. Severe sepsis was defined as infection and acute organ dysfunction. Clinical and laboratory variables used in clinical routines were included for screening. Subjects without missing values were included, and the whole dataset was split into training and validation cohorts. The score was coined LASSO score because variable selection was performed using the least absolute shrinkage and selection operator (LASSO) technique. Finally, the LASSO score was evaluated for its discrimination and calibration in the validation cohort.

**Conclusions:**

The study developed the LASSO score for mortality prediction in patients with severe sepsis. Although the score had good discrimination and calibration in a randomly selected subsample, external validations are still required.

## INTRODUCTION

Severe sepsis is one of the leading cause of morbidity and mortality in the intensive care unit (ICU). Despite strenuous effort to improve its survival, the mortality rate remains unacceptably high [1–3]. A priority in the management of severe sepsis is to predict its outcome, which can further help to triage patients and inform family members. A variety of prognosticating scores have been developed and validated in external cohorts for their discrimination and calibration. Among them, the sepsis-related organ failure assessment (SOFA) score received most attentions. Although SOFA score was simple to use and accurate in predicting mortality outcome, it was developed based on expert consensus [4]. Laboratory and treatment variables chosen for each organ system were somewhat arbitrary, and some empirical evidence showed that some component variables were not linearly associated with mortality in Logit scale and the cutoff points were arbitrary. For example, the hepatic score showed no independent contribution to the mortality risk [5, 6].

Conventional method to develop illness severity score was first to screen variables that were associated with mortality by bivariate analysis. However, this method has been criticized for its limitations because covariates of high correlation can be included [7]. The least absolute shrinkage and selection operator (LASSO) was developed in 1996, which was particularly suitable for variable selection among large amount of variables for the prediction of an outcome [8]. Prediction in severe sepsis is such a situation that a large amount of laboratory and treatment variables are collected. With LASSO method, coefficients of unimportant variables will be penalized to zero while important variables will be retained. The present study aimed to develop a new scoring system for the prognostication of severe sepsis. The new score was termed LASSO score because the LASSO technique was employed to train the model.

## RESULTS

**Figure 1:**
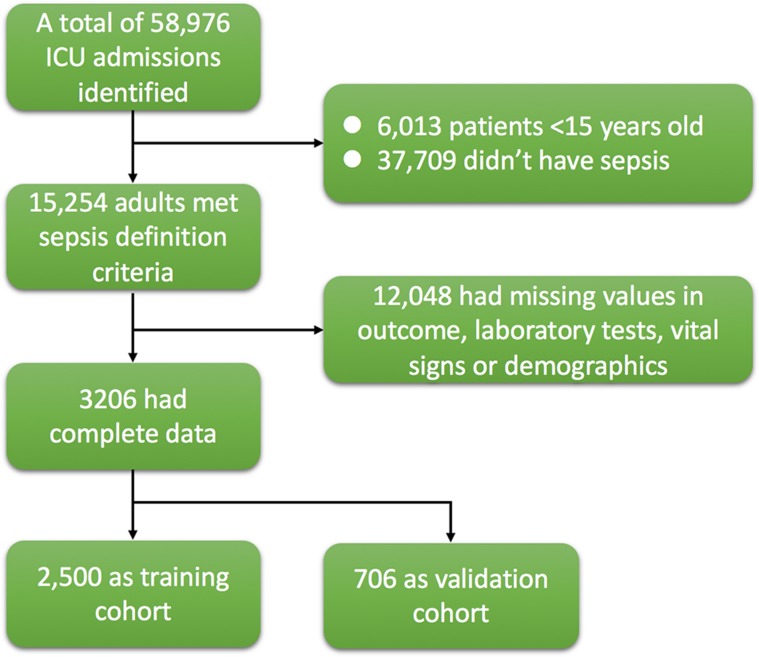
Flowchart of patient selection There were 3206 patients with complete data being used for analysis, including 1054 non-survivors and 2152 survivors.

The initial search identified 58,976 ICU admissions in the MIMIC-III database. 15,254 of them were adult and met the criteria of severe sepsis. There were 3206 patients with complete data (Figure 1), including 1054 non-survivors and 2152 survivors. Comparisons of demographics and variables were shown in Supplementary Tables 1 and 2. Non-survivors were older than survivors (80 ± 58 vs. 70 ± 48 years; *p* < 0.01). Non-survivors had significantly higher A-aO2 (289.62 ± 141.16 vs. 235.04 ± 128.83 mmHg; *p* < 0.01), bilirubin (3.35 ± 5.98 vs. 1.83 ± 4.34 mg/dl; *p* < 0.01), BUN (44.47 ± 28.05 vs. 34.30 ± 25.05 mg/dl; *p* < 0.01), heart rate (97 ± 19 vs. 94 ± 18 /min; *p* < 0.01), INR (2.05 ± 1.58 vs. 1.64 ± 1.25; *p* < 0.01), lactate (4.03 ± 3.34 vs. 2.46 ± 1.70 mmol/l; *p* < 0.01), aPTT (48.1 ± 22.9 vs. 40.5 ± 19.5 s; *p* < 0.01), respiratory rate (22 ± 5 vs. 21 ± 5 /min; *p* < 0.01), WBC (15.7 ± 18.7 vs. 14.0 ± 8.4; *p* = 0.005) than those in survivors. Survivors showed significantly higher albumin (2.91 ± 0.65 vs. 2.66 ± 0.68 mg/dl; *p* < 0.01), bicarbonate (22.55 ± 5.43 vs. 20.38 ± 5.94 mmol/l; *p* < 0.01), diastolic blood pressure (63.85 ± 11.75 vs. 59.58 ± 12.71 mmHg; *p* < 0.01), pH (7.35 ± 0.08 vs. 7.31 ± 0.10; *p* < 0.01), platelet (229.6 ± 136.3 vs. 192.8 ± 138.6; *p* < 0.01), PaO2 (166.5 ± 68.8 vs. 160.2 ± 71.1 mmHg; *p* = 0.02), systolic blood pressure (119 ± 16 vs. 111 ± 17 mmHg; *p* < 0.01), body temperature (37.0 ± 0.8 vs. 36.7 ± 1.0°C; *p* < 0.01), GCS (13.3 ± 3.2 vs. 13.0 ± 3.0; *p* = 0.02), urine output (1750 ± 1652 vs. 1078 ± 1134 ml/24 h; *p* < 0.01) than those in non-survivors. Higher doses of dopamine (2.7 ± 7.0 vs. 1.4 ± 8.4 mg/kg/min; *p* < 0.01), epinephrine (0.01 ± 0.08 vs. 0.003 ± 0.02 mg/kg/min; *p* = 0.012), norepinephrine (0.2 ± 0.4 vs. 0.1 ± 1.4 mg/kg/min; *p* = 0.049) and dobutamine (0.3 ± 1.8 vs. 0.2 ± 1.1 mg/kg/min; *p* = 0.009) were associated with increased risk of hospital death. Mechanical ventilation was associated with increased risk of death (1002 [95%] vs. 1978 [92%]; *p* = 0.001). Patients with cardiac arrhythmia (330 [31%] vs. 555 [26%]; *p* = 0.001), lymphoma (37 [4%] vs. 30 [1%]; *p* < 0.01) and metastatic cancer (95 [9%] vs. 81 [4%]; *p* < 0.001) showed elevated risk of death. Obesity was associated with reduced risk of death (49 [5%] vs. 180 [8%]; *p* < 0.001).

**Figure 2:**
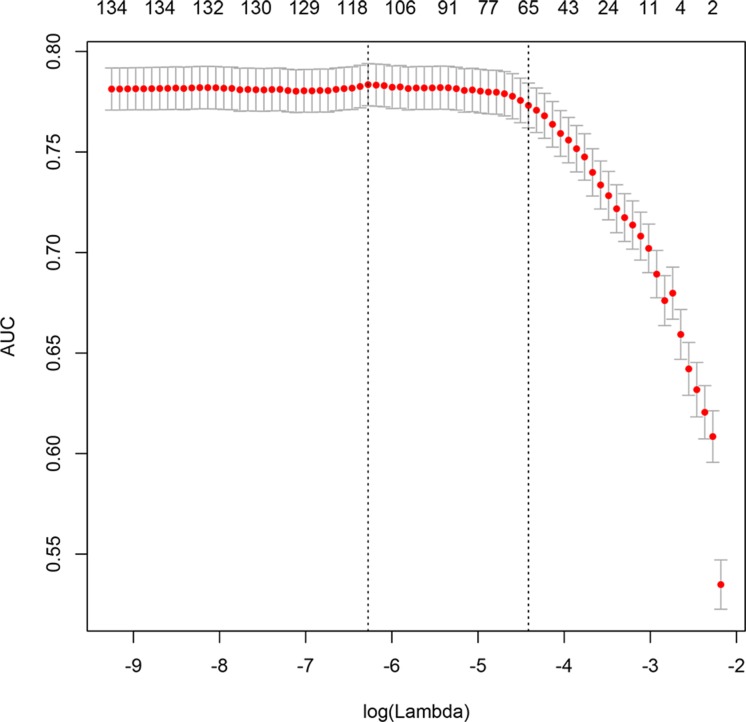
Different area under curve (AUC) values across the range of lambda The AUC was estimated with cross-validation technique and the largest lambda value was chosen when the cross-validation error was within one standard error of the minimum.

**Figure 3:**
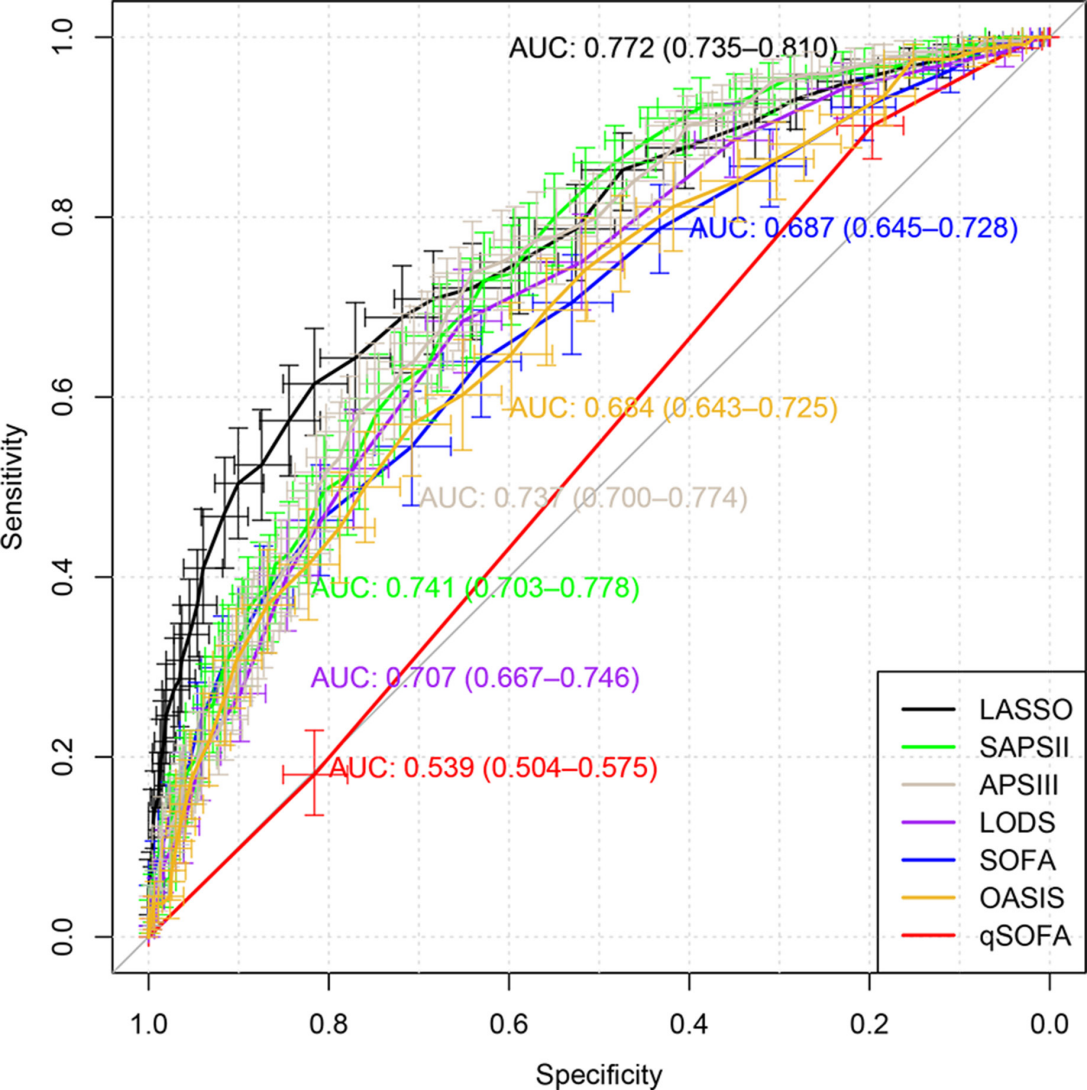
Discrimination of the LASSO score was evaluated in the validation cohort and compared with other scores

**Figure 4:**
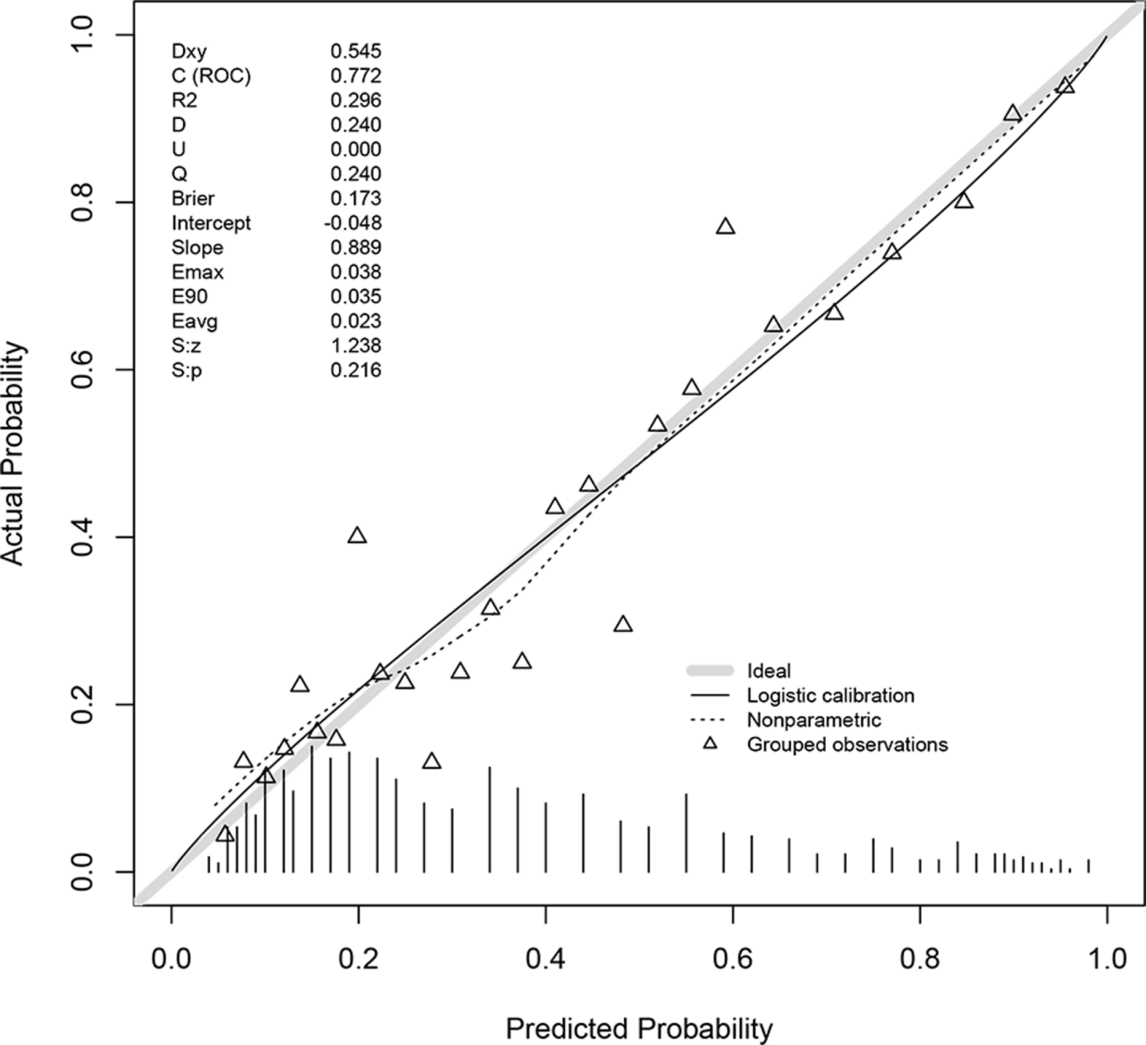
Calibration of LASSO score in the validation cohort The calibration slope was 0.889 and the Brier value was 0.173.

**Figure 5:**
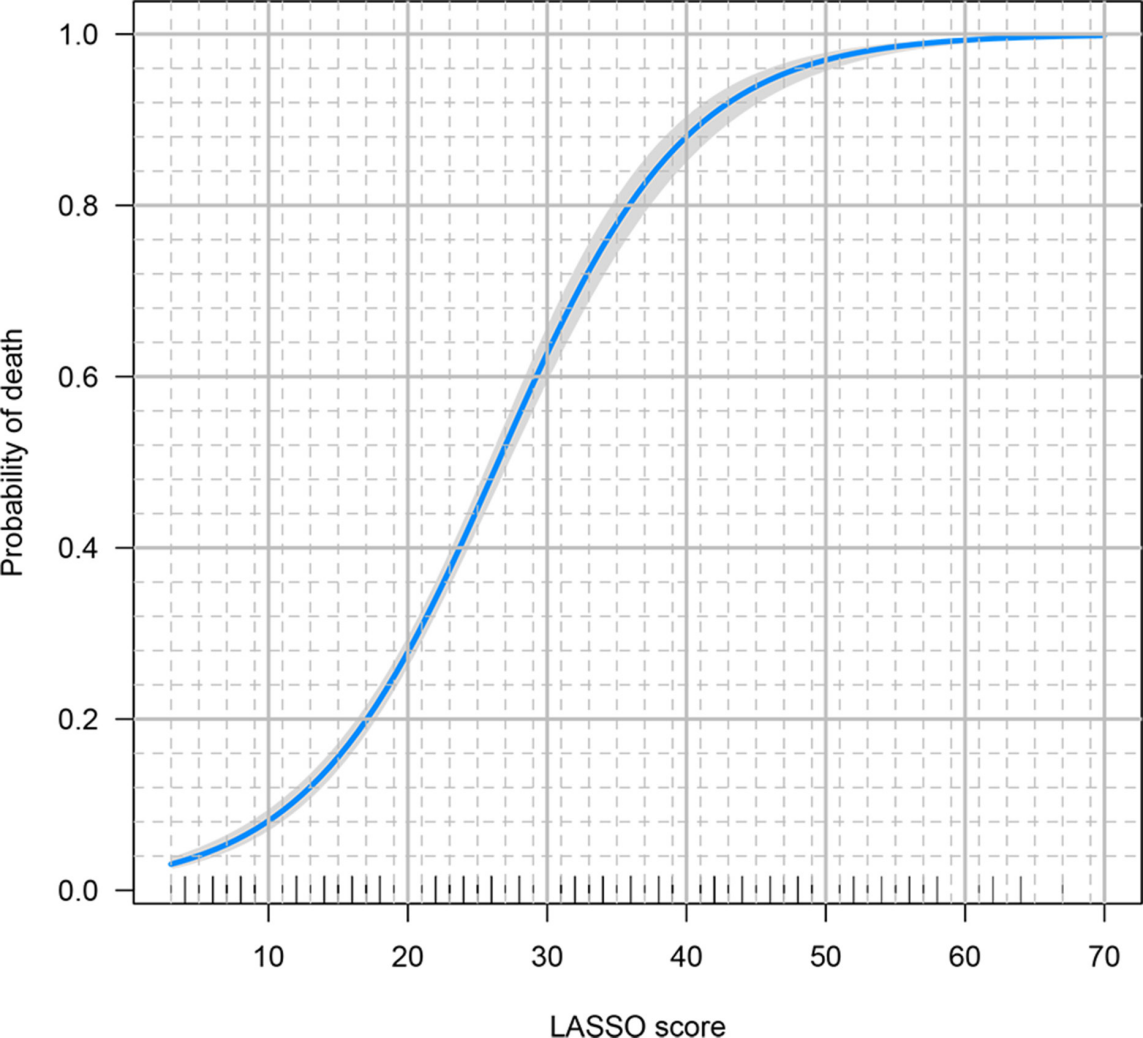
The relationship between LASSO score and probability of death The gray area is the 95% confidence interval of the prediction. At a LASSO score of 30, the probability of death was approximately 60%.

Loess smoothing curves for continuous variables are shown in Supplementary Figures 1–38. Figure 2 shows different AUC values across the range of lambda. The AUC was estimated with cross-validation technique and the largest lambda value was chosen when the cross-validation error was within one standard error of the minimum. It appeared that 65 dummy variables were retained in the model. Points assigned to each range of continuous variable and categories were shown in Supplementary Table 3. Note that the presence of obesity was assigned -2 points because it was a protective factor. Discrimination of the LASSO score was evaluated in the validation cohort and compared with other scores (Figure 3). The LASSO score showed the largest discrimination ability (AUC: 0.772; 95% CI: 0.735-0.810), followed by SAPSII (AUC: 0.741; 95% CI: 0.703-0.778), APSIII, (AUC: 0.737; 95% CI: 0.700-0.774) and LODS (AUC: 0.707; 95% CI: 0.667-0.746). The LASSO score showed significantly better discrimination than the SOFA score (AUC: 0.687; 95% CI: 0.643-0.725). The calibration of LASSO score in the validation cohort was shown in Figure 4. The calibration slope was 0.889 and the Brier value was 0.173. Figure 5 shows the relationship between LASSO score and probability of death. The gray area is the 95% confidence interval of the prediction. At a LASSO score of 30, the probability of death was approximately 60%.

## DISCUSSION

The study developed a new scoring system for severe sepsis. It was coined LASSO score because the LASSO technique was employed to develop the score. Discrimination and calibration of the score were evaluated in the validation cohort and the result showed that the discrimination was comparable to other scores such as SAPSII, APSIII and LODS. However, the discrimination of LASSO was significantly better than SOFA.

There are a variety of scores being used for the triage of critically ill patients, but only SOFA has been proposed to be used specifically for sepsis [4]. However, SOFA was originally developed by expert consensus and validated in subsequent dataset. Although SOFA score has been extensively validated in subsequent studies, its discriminations varied substantially due to case mix [9]. The reported AUC of SOFA score measured on admission for mortality prediction ranged from 0.61 to 0.88 [6, 9, 10]. The components of SOFA score are related to organ dysfunctions and some important prognosticators are not included. A large body of evidence have shown that obesity was a protective factor for critically ill patients, and the effect is robust across several subgroups such as severe sepsis, acute respiratory distress syndrome and cardiac surgery [11–14]. In a meta-analysis involving six studies [13], overweight and obesity were associated with reduced odds ratio of mortality [0.83 (0.75, 0.91) *p* < 0.001 and 0.82 (0.67, 0.99) *p* = 0.04, respectively]. In our study, we used robust LASSO technique and the obesity remain consistently to be a protective factor. The LASSO score included obesity as a component and assigned minus 2 points to it. Similarly, numerous studies have found that serum lactate was an strong independent predictor of mortality [15–17], but none of the above mentioned scores included it. Since the measurement of lactate has become a clinical routine, it is time to add it as a component of severity score. The LASSO score assigned 10 points to lactate, which was in line with previous finding that lactate was a strong and independent predictor of mortality [18, 19].

There were limitations in the present study. The study used dataset from a single center. Although cross-validation was used for selection of lambda value and the LASSO score was validated in the validation dataset, the importance of external validation can never be overstated [20]. Further studies are required to evaluate the discrimination and calibration of LASSO score in external datasets. For many continuous variables, there were limited number of subjects at both ends of the whole range. Thus, points assigned to extreme values may not be stable. In the study we combine the extreme values into their next intervals. Severe sepsis was defined as infection and acute organ dysfunction, and acute organ dysfunctions were identified according to ICD-9 in electronic healthcare records. Such a definition is different from the one that has been recommended in the Sepsis-3.0 [21]. However, there is no formal diagnosis for the severe sepsis in the ICD system and we had to implement data manipulation to identify patients who had documented infection and acute organ failure. Another limitation of the study referred to the definition of routine clinical variables. The definition of “routine” may vary widely among different hospitals and institutions. However, in the study we included all variables that have been investigated in establishing other severity scores [9, 22, 23], as well as those that were found to be relevant in predicting mortality outcome such as obesity and serum lactate [11, 13, 16, 24]. Lastly, the timing of variable measurement is somewhat arbitrary. The timing was determined in accordance with other severity scores [4, 6, 22]. If the sampling time is too early, many laboratory variables are not reported, compromising the ability of the predictive performance. On the other hand, if the sampling time is late, the predictive accuracy can be improved because variables are measured close to the occurrence of the outcome, but the timeliness of the prediction is compromised. The use of 24 hours after ICU admission is a trade-off between timeliness and prediction accuracy.

In conclusion, the study developed the LASSO score for mortality prediction in patients with severe sepsis. The score had good discrimination and calibration in a randomly selected subsample. Further studies are required to validate the score in external cohorts.

## MATERIALS AND METHODS

### Database

The study employed publically available database Medical Information Mart for Intensive Care-III) MIMIC-III for analysis. Details of the database were fully described elsewhere [25]. Briefly, MIMIC-III comprised ICU patients admitted to the Beth Israel Deaconess Medical Center in Boston, Massachusetts from the year 2001 to 2012. It contained more than 50,000 adult distinct ICU admissions and nearly 8,000 neonates admissions. Data on laboratory tests, demographics, drugs, diagnosis, microbiology findings and survival status on hospital discharge were available. The establishment of the database was approved by the Massachusetts Institute of Technology (Cambridge, MA) and the Institutional Review Boards of Beth Israel Deaconess Medical Center (Boston, MA). Requirement for individual patient consent was waived because the project did not impact clinical care and all protected health information was deidentified [25].

### Study population

Severe sepsis was defined as infection and acute organ dysfunction [26, 27], which had been described elsewhere [28]. In the electronic healthcare records, a subject was defined to have infection if his or her ICD-9 code indicated a diagnosis of infection. ICD-9 code for acute organ failure included Hypotension (458), Acute and subacute necrosis of liver (570), Transient mental disorders due to conditions classified elsewhere (293), Shock without mention of trauma (7855), Anoxic brain damage (3481), Secondary thrombocytopenia (2874), Encephalopathy (3483), unspecified thrombocytopenia(2875), Other and unspecified coagulation defects (2869), Defibrination syndrome (2866), acute kidney failure (584) and Hepatic infarction (5734). If mechanical ventilation (procedures ICD code: 9670, 9671, 9672) was required, it was also defined as organ dysfunction. Patients were excluded if 1) they were younger than 16 years old; or 2) variables contained missing values.

### Variables included in the analysis

Clinical and laboratory variables that were used in routine clinical practice were included for screening. Demographic data included age, gender, admission type (emergency, post-operative, ward), and ethnicity. Laboratory variables include alveolar–arterial gradient (A-aO_2_), albumin, bicarbonate, bilirubin, blood urea nitrogen (BUN), coagulation profile, partial pressure of arterial carbon dioxide (PaCO_2_), partial pressure of arterial oxygen (PaO_2_), lactate, pH, lactate, platelet, electrolytes, total CO_2_, and white blood cell (WBC) count. Vital signs included heart rate, systolic blood pressure, mean arterial blood pressure, diastolic blood pressure, body temperature, and respiratory rate. Comorbidities included obesity, cardiac arrhythmia, lymphoma and metastatic cancer. Vasopressors and inotropes included dopamine, norepinephrine, epinephrine and dobutamine. Other variables included Glasgow Coma scale (GCS) and urine output. All variables were recorded during the first 24 hours after ICU admission. If there were several measurements, the worst one was included for the analysis. For continuous variables, the worst one may be the lowest or the highest value, we used the one with the highest points in other severity scores. All continuous variables except for the lactate had been used in other severity scores such as simplified acute physiology score (SAPS) II, acute physiological score (APS) III, Logistic organ dysfunction system (LODS), SOFA, Oxford Acute Severity of Illness Score (OASIS) and quick SOFA (qSOFA) [22, 23, 29–32].

Outcome variable was the vital status at hospital discharge. There were survivors and non-survivors. Admissions with missing information on outcome were excluded.

### Statistical analysis

Continuous variables were expressed as mean and standard deviation (SD), and compared between survivors and non-survivors with *t* test. Categorical variables were expressed as the number and proportion, and were compared between groups with Chi-square test [33].

The complete dataset was randomly split into training and validation datasets. The training dataset comprised 78% of the total subjects, and the remaining constituted the validation cohort. Continuous variables were cut into several intervals, with each interval corresponding to a certain range of mortality risk. Firstly, continuous variables were plotted against vital status at hospital discharge and Loess smoothing function was fit to suggest the cutoff points for each variable [34]. Cutoff points corresponding to the probability of death at 0.2, 0.3, 0.4, 0.5, 0.6 and 0.7 were used. One continuous variable was made into dummy variables by the cutoff points. Then, dummy variables and categorical variables were entered into logistic regression model. Variable selection was performed by using the LASSO technique. Briefly, the technique shrunk coefficient estimates towards zero by tuning parameter lambda. Similar to the best subset selection, LASSO technique forces some of the coefficient estimates to be exactly zero when the lambda is sufficiently large. The largest value of lambda was chosen for which the cross-validation error was within one standard error of the minimum [35].

The LASSO regression model would return coefficients for each variables, and these coefficients were used to assign points to each range. The coefficient of each range was multiplied by 10 and rounded off to the nearest integer. Thereafter, the LASSO score for each patient could be calculated. A logistic regression model would be built to convert this score to the probability of hospital mortality.

The scoring system was validated in the validation dataset. Also the discrimination of LASSO score was compared with other commonly used scores such as SAPS-II, APS-III, LODS, SOFA, OASIS and qSOFA in the validation cohort. Discrimination, which measures the ability of the score to correctly classify hospital survivors and non-survivors, was represented by the area under receiver operating characteristic (ROC) curve [36]. Calibration of LASSO in the validation cohort was also evaluated. All statistical analyses were performed using R (version 3.3.2).

## SUPPLEMENTARY MATERIALS FIGURES AND TABLES




