# Supplementary material for: Development of a novel score for the prediction of hospital mortality in patients with severe sepsis: the use of electronic healthcare records with LASSO regression
Source: Oncotarget. 2017 May 15;8(30):49637–45. doi: 10.18632/oncotarget.17870 (PMC5564794; doi:10.18632/oncotarget.17870)
Supplement: Supplementary file 1 [file oncotarget-08-49637-s001.pdf]

Development of a novel score for the prediction of hospital mortality in patients with severe sepsis: the use of electronic healthcare records with LASSO regression

Supplementary Materials

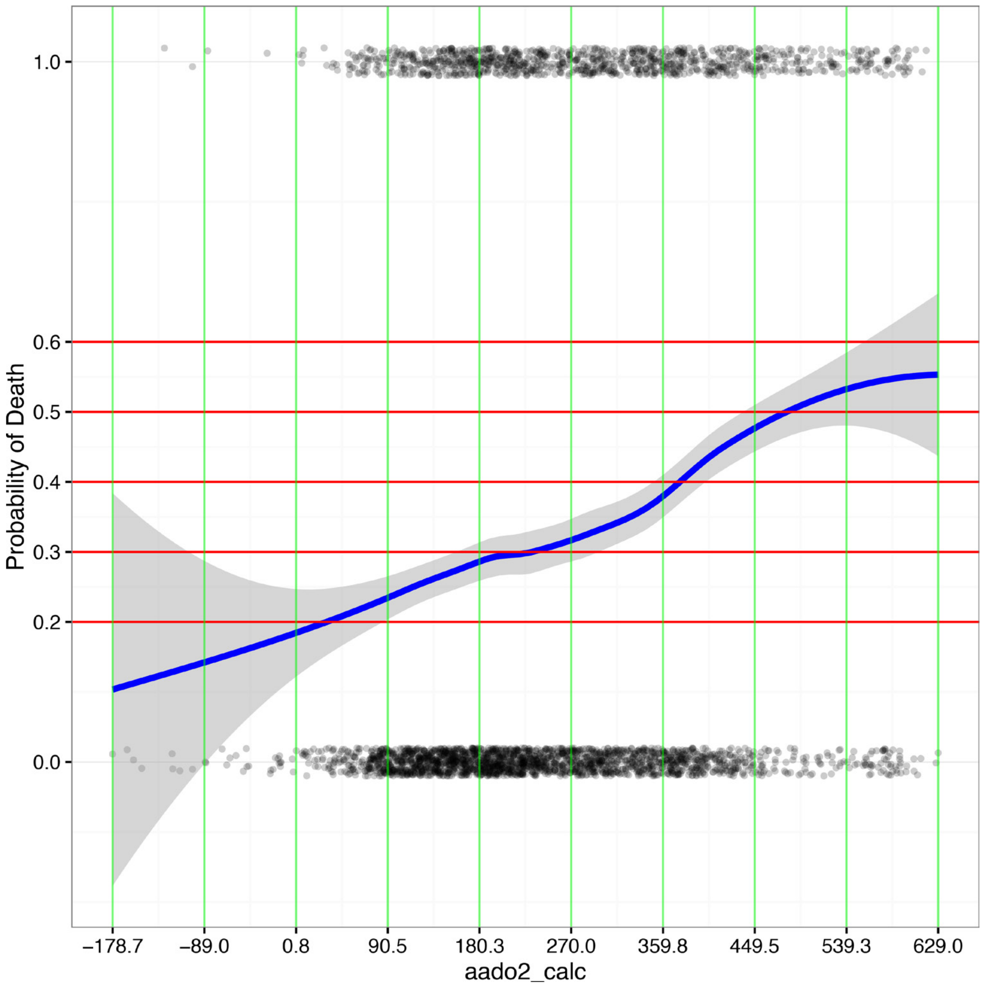

Supplementary Figure 1: Loess smoothing curve showing the association of Alveolar–arterial gradient with risk of death

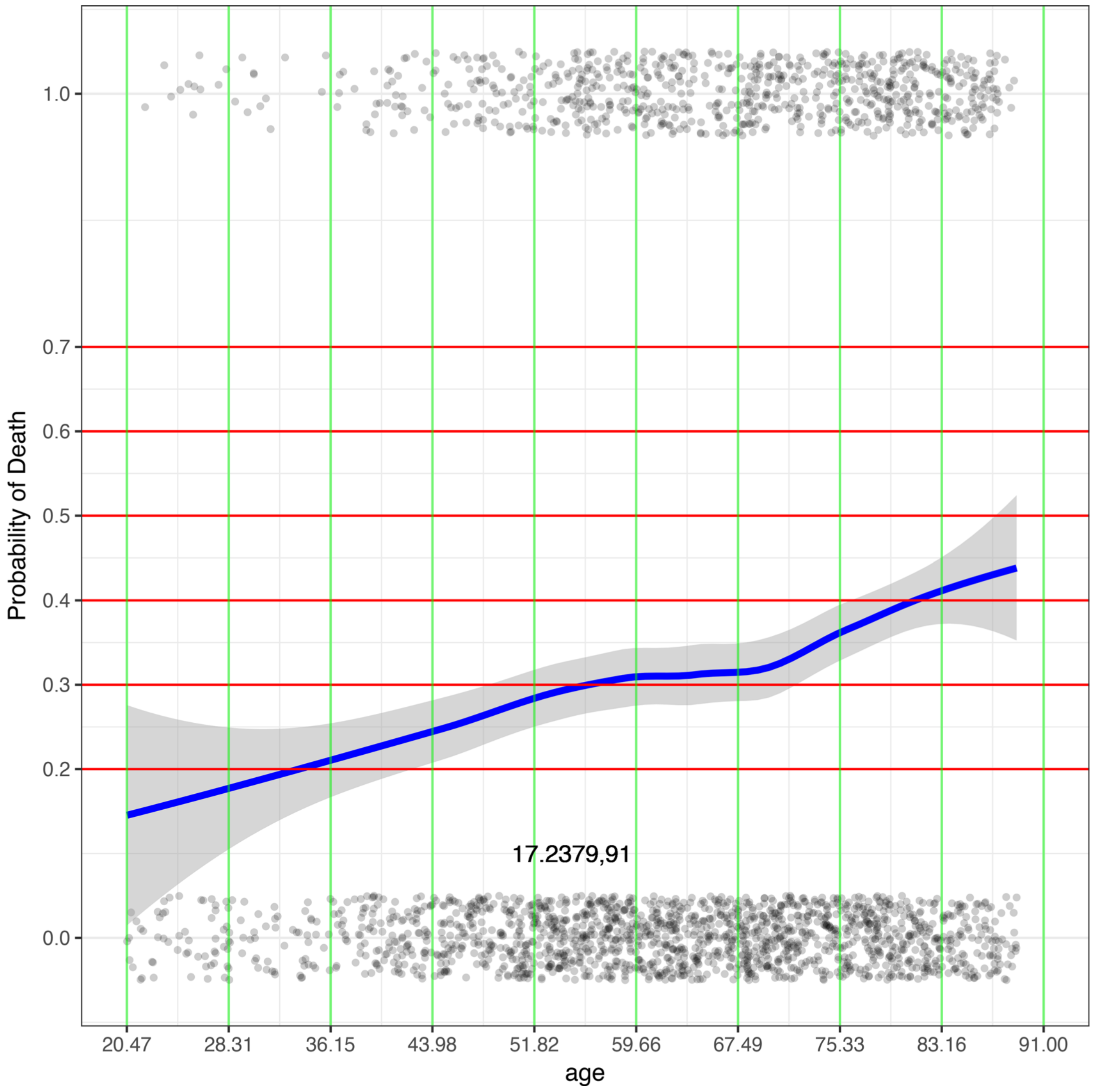

Supplementary Figure 2: Loess smoothing curve showing the association of age with the risk of death

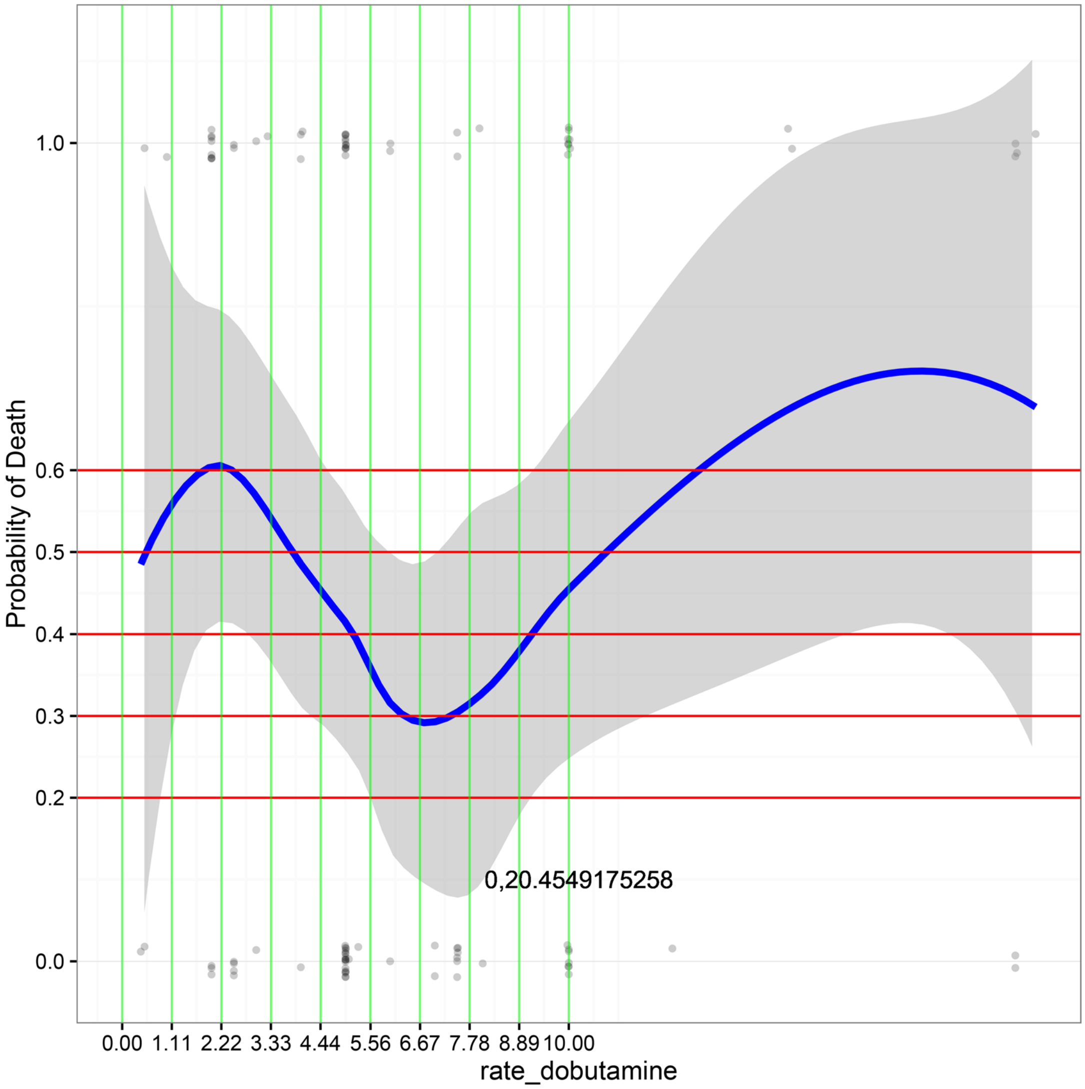

Supplementary Figure 3: Loess smoothing curve showing the association of dobutamine dosage with the risk of death

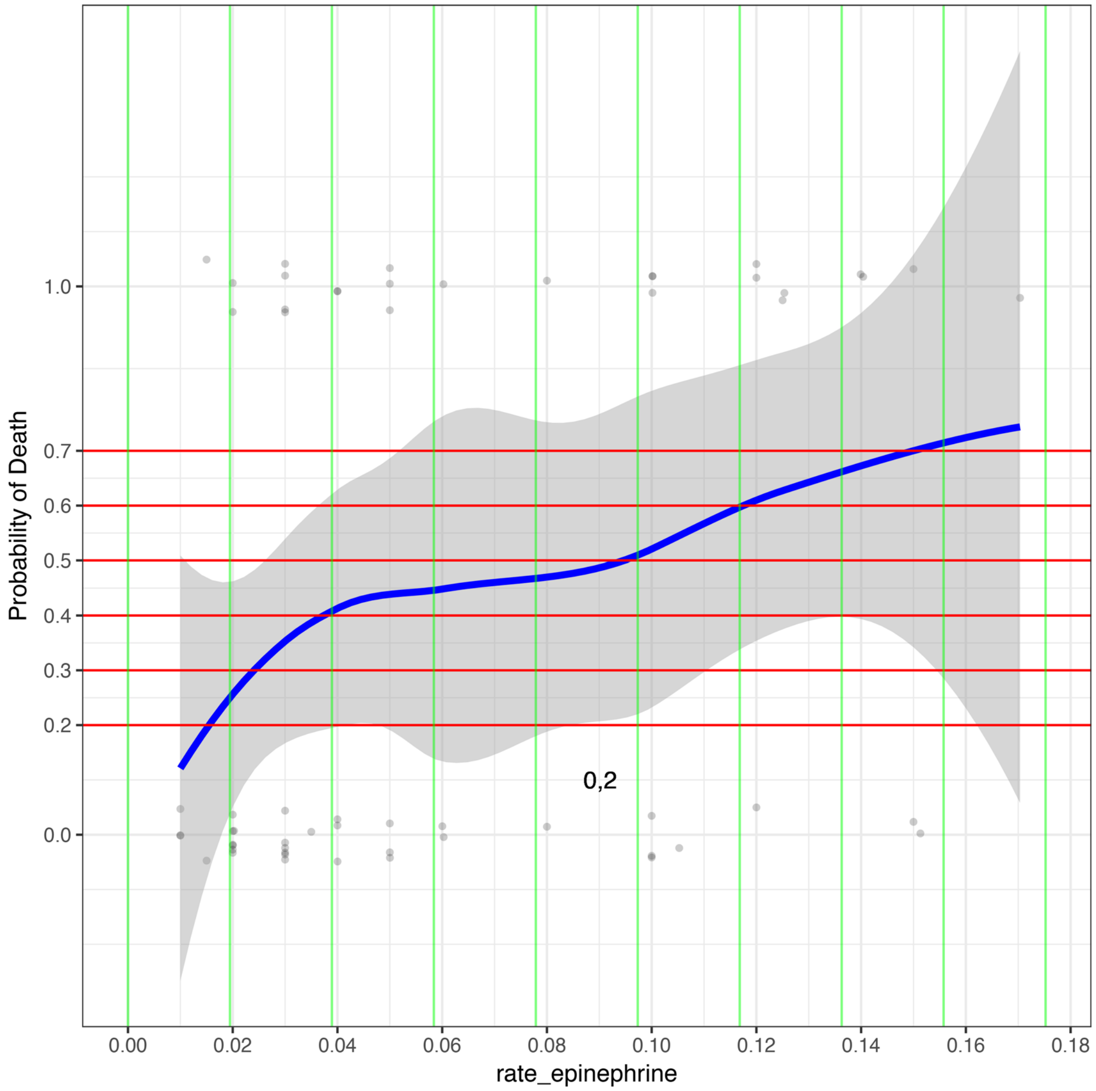

Supplementary Figure 4: Loess smoothing curve showing the association of epinephrine dosage with the risk of death

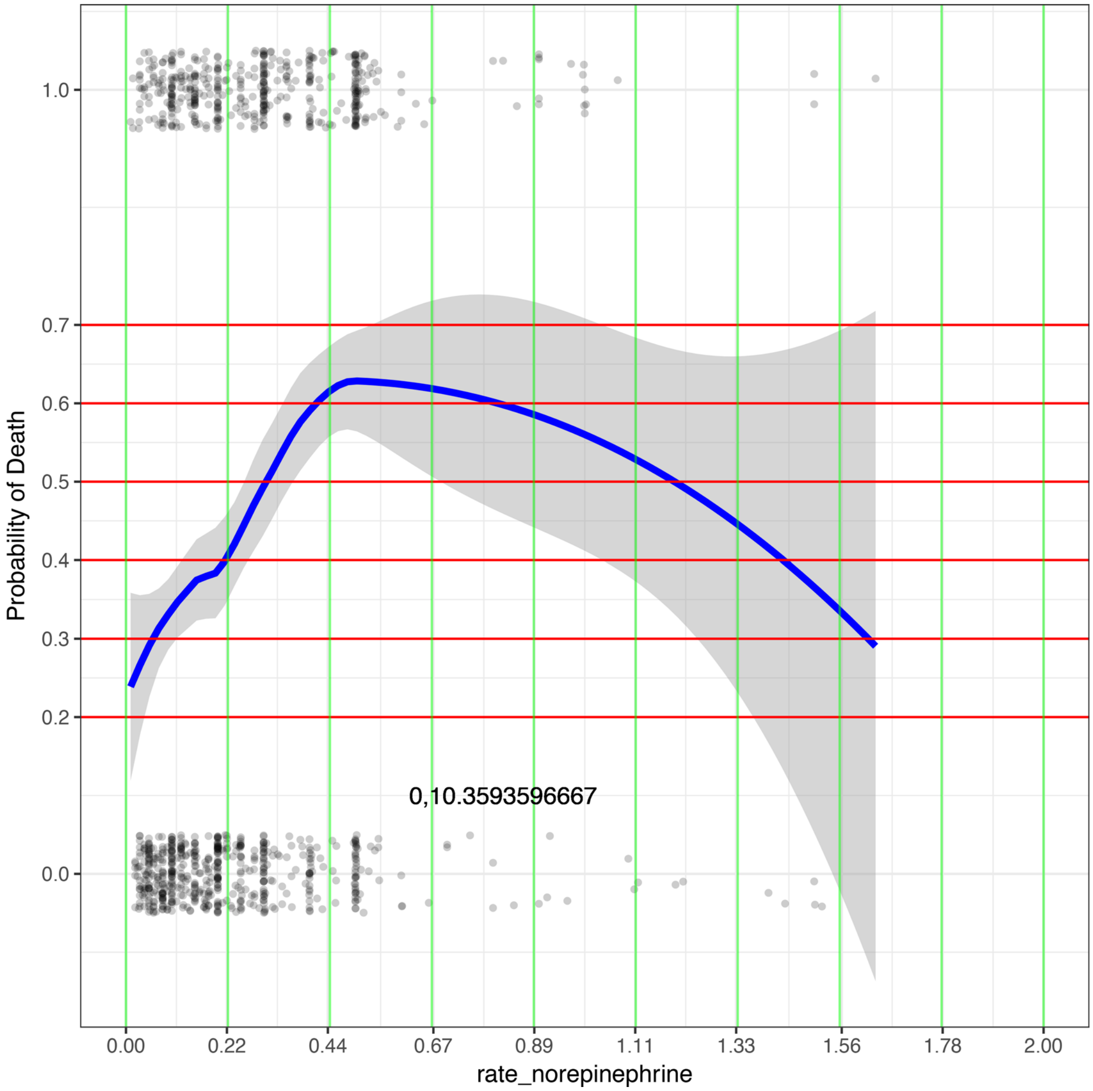

Supplementary Figure 5: Loess smoothing curve showing the association of norepinephrine dosage with the risk of death

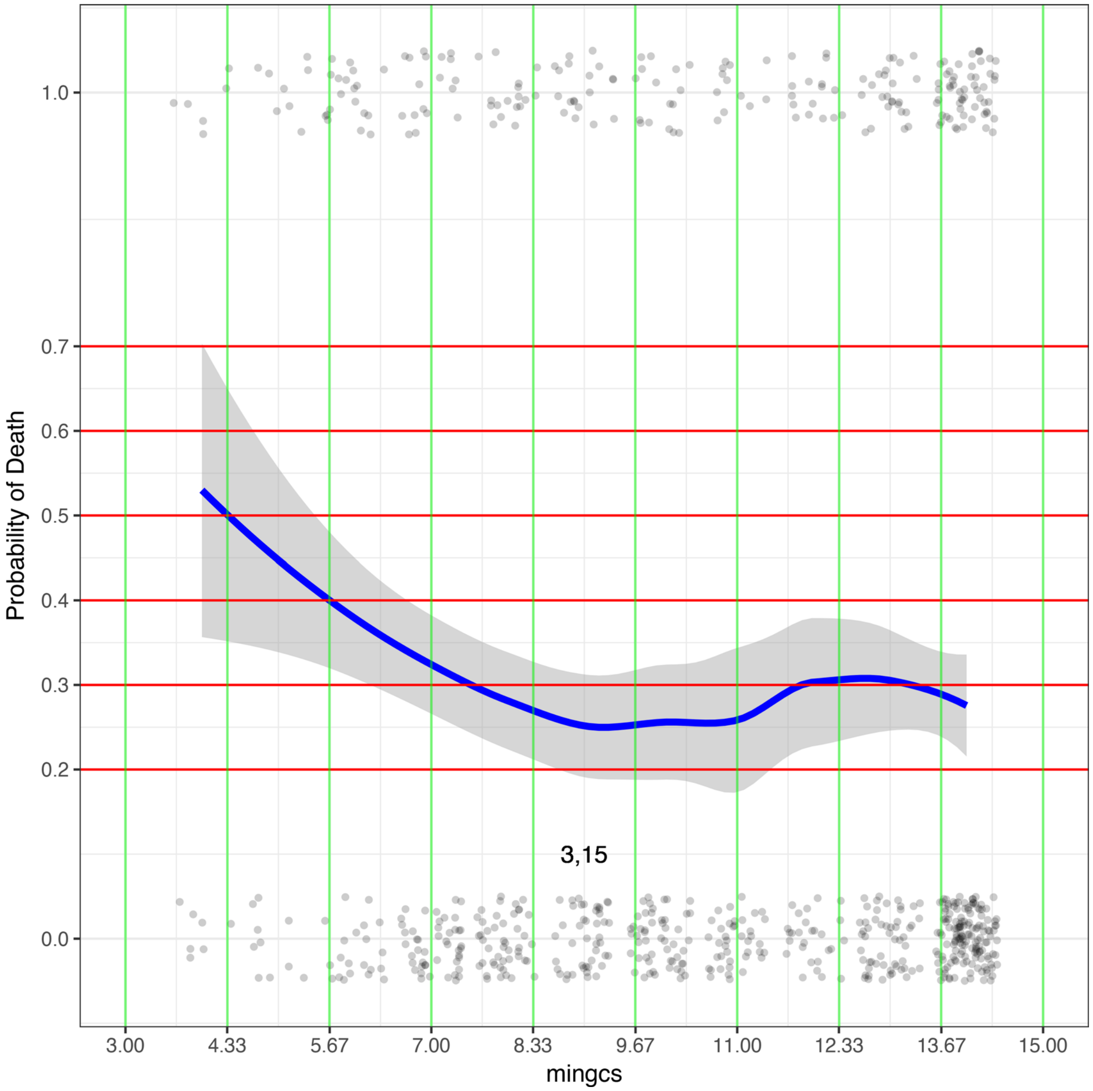

Supplementary Figure 6: Loess smoothing curve showing the association of minimum GCS with the risk of death

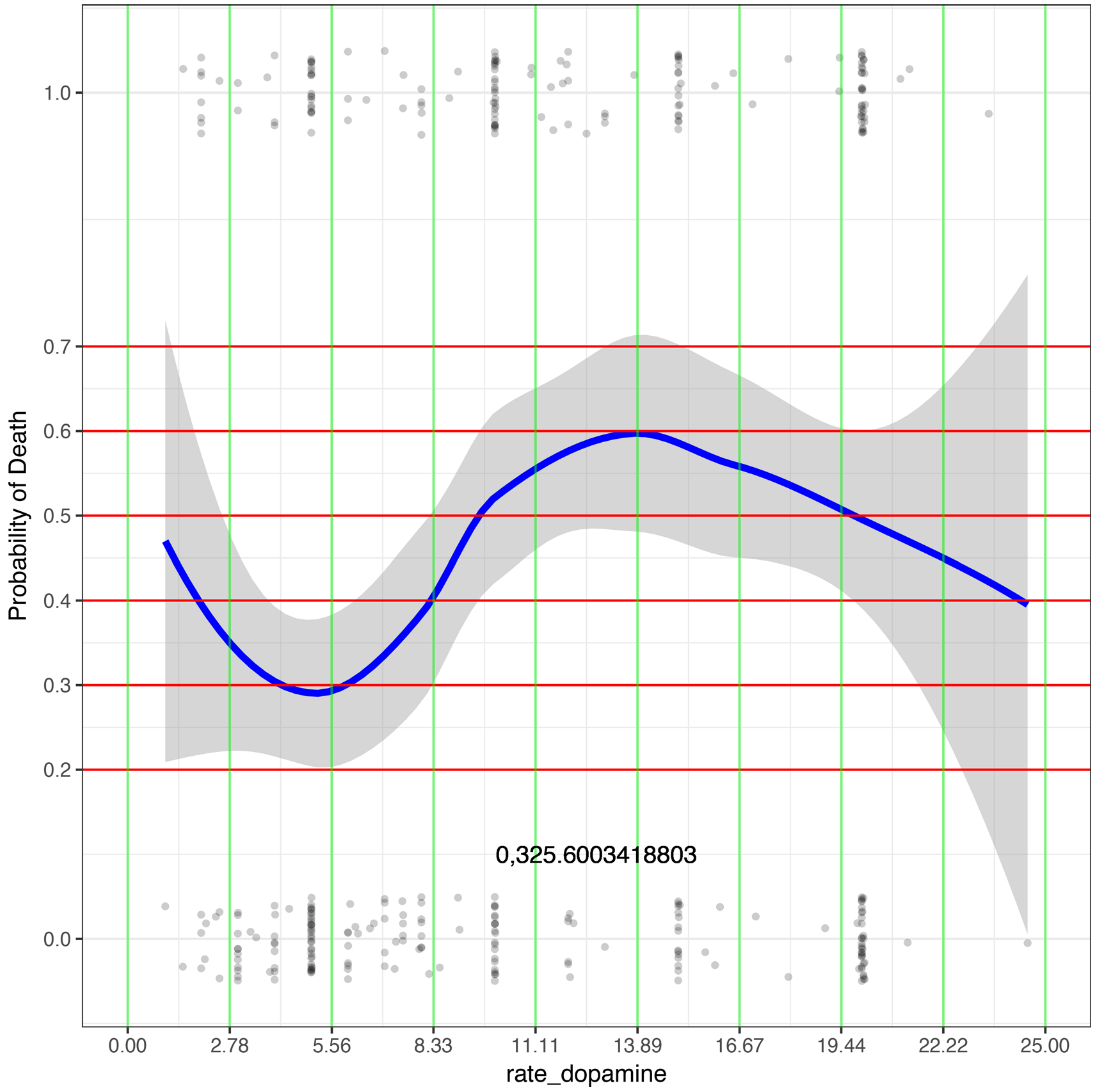

Supplementary Figure 7: Loess smoothing curve showing the association of dopamine with the risk of death

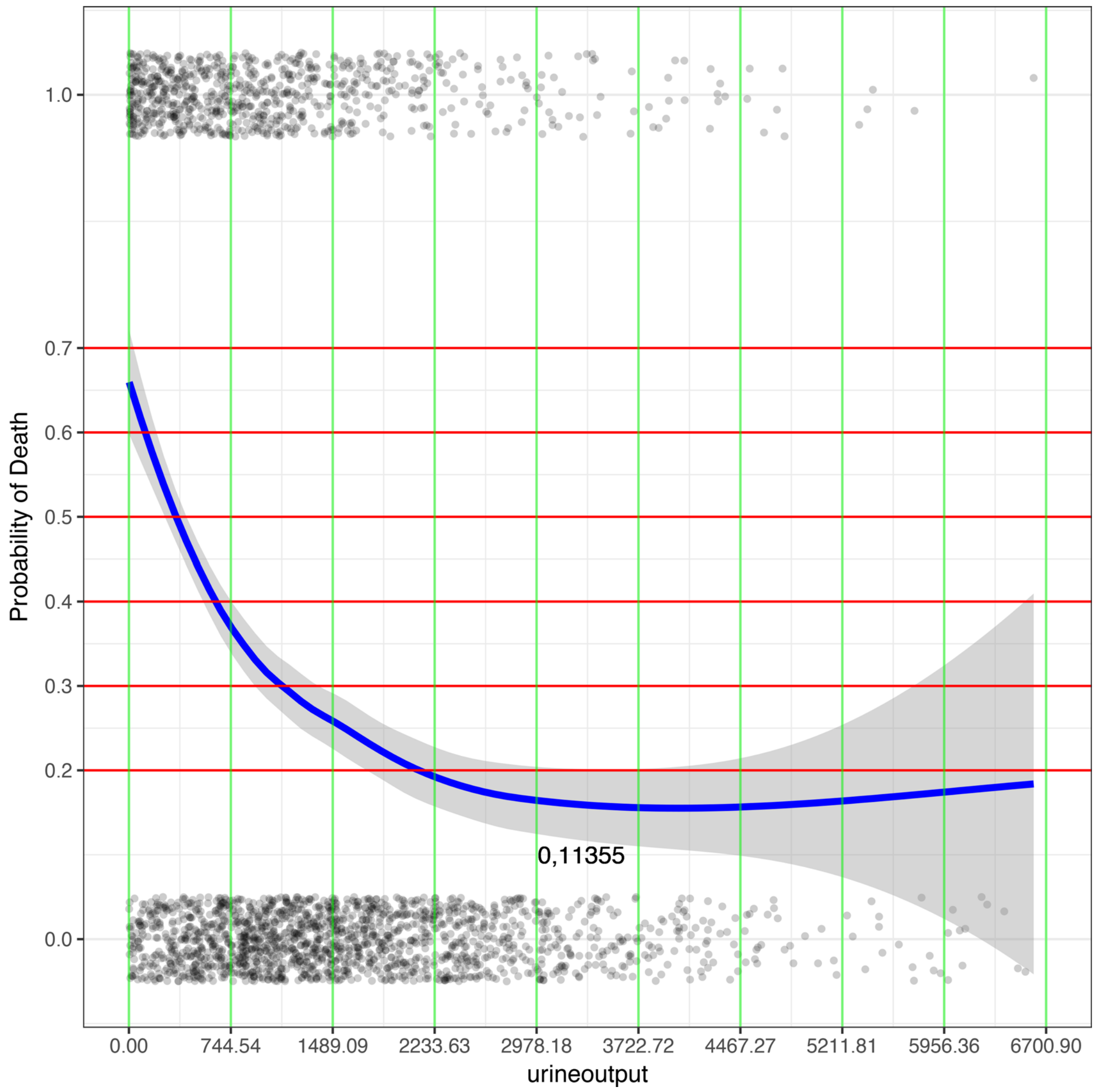

Supplementary Figure 8: Loess smoothing curve showing the association of urine output with the risk of death

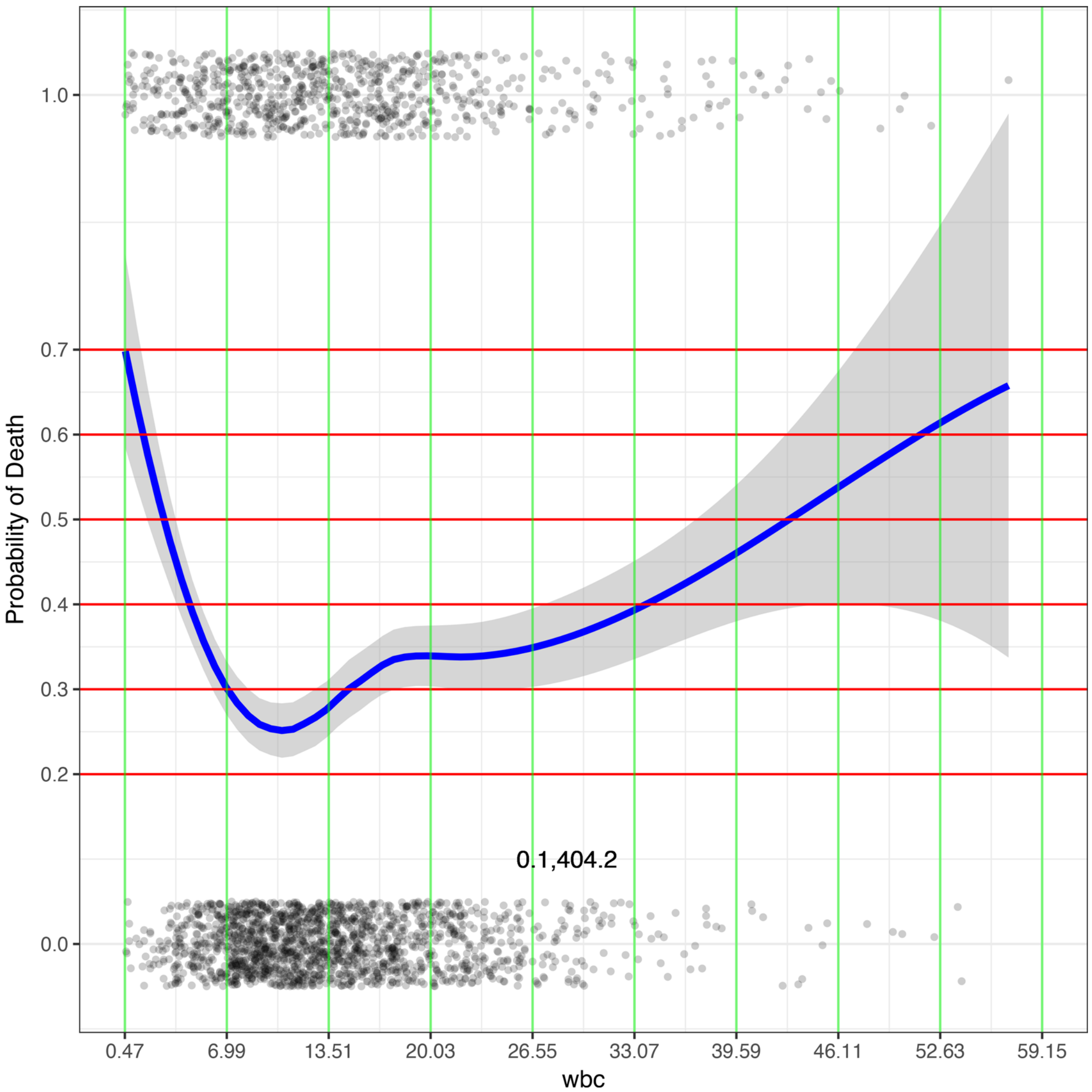

Supplementary Figure 9: Loess smoothing curve showing the association of white blood cell count with the risk of death

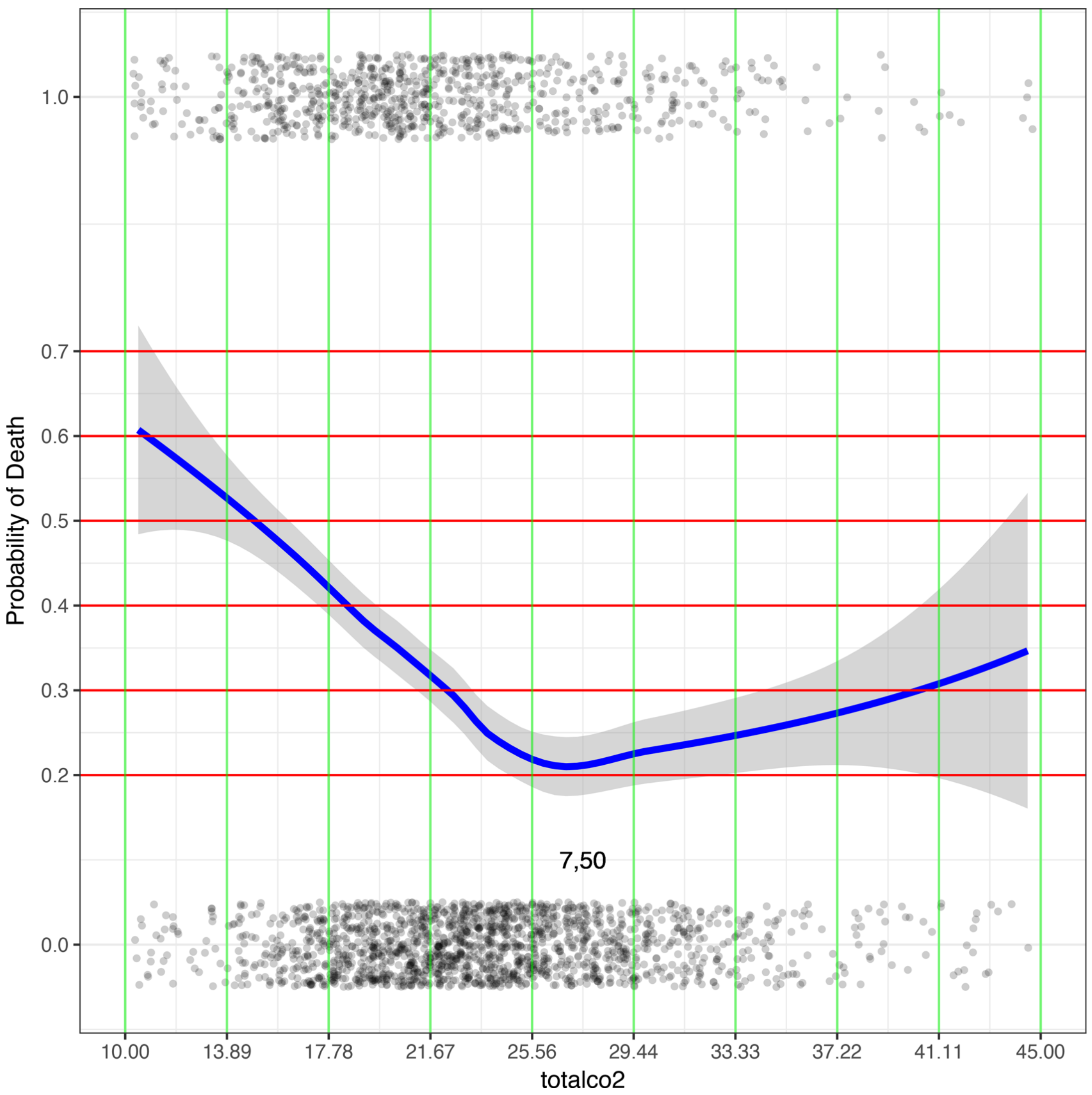

Supplementary Figure 10: Loess smoothing curve showing the association of total CO2 with the risk of death

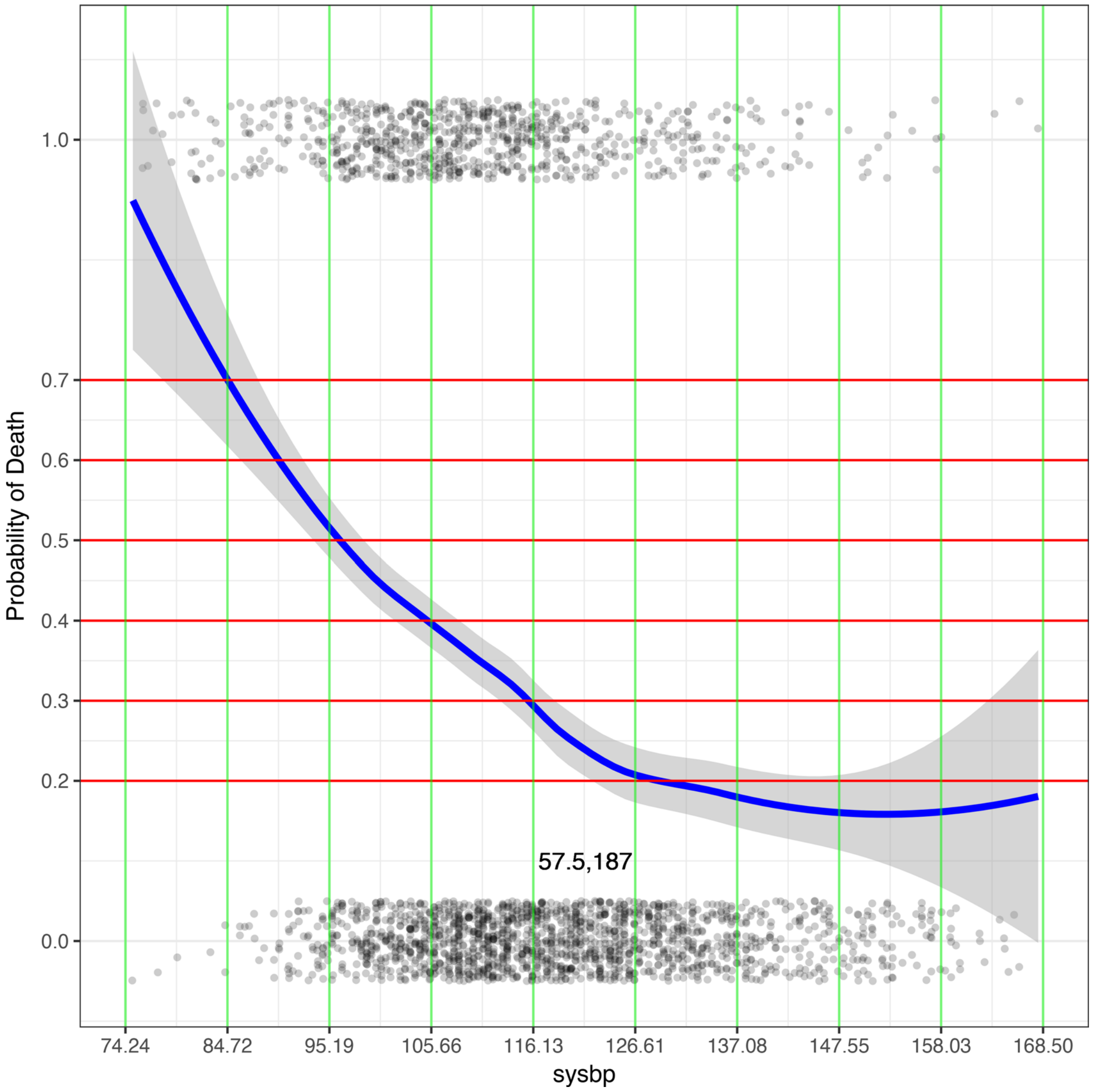

Supplementary Figure 11: Loess smoothing curve showing the association of systolic blood pressure with the risk of death

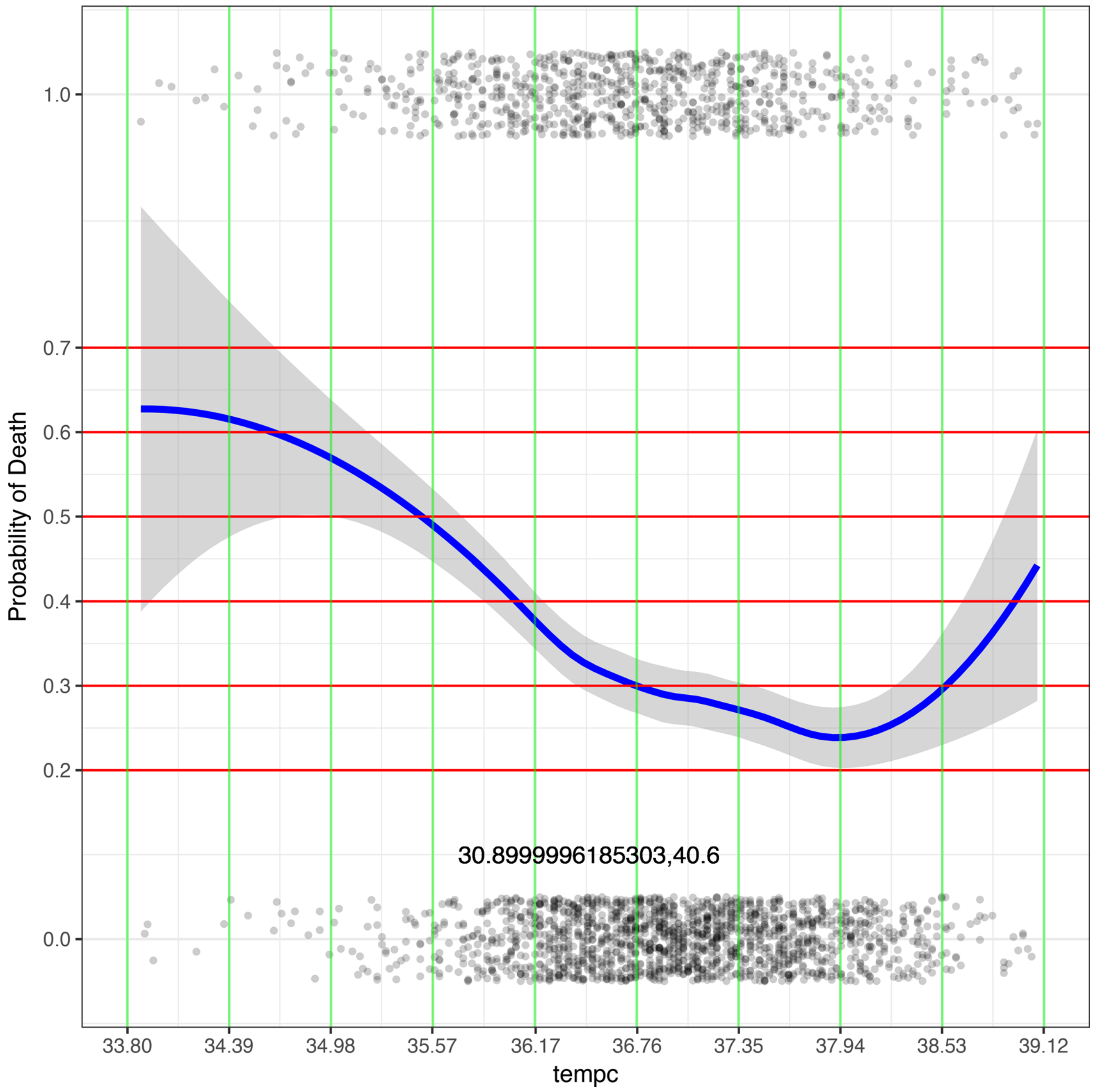

Supplementary Figure 12: Loess smoothing curve showing the association of body temperature with the risk of death

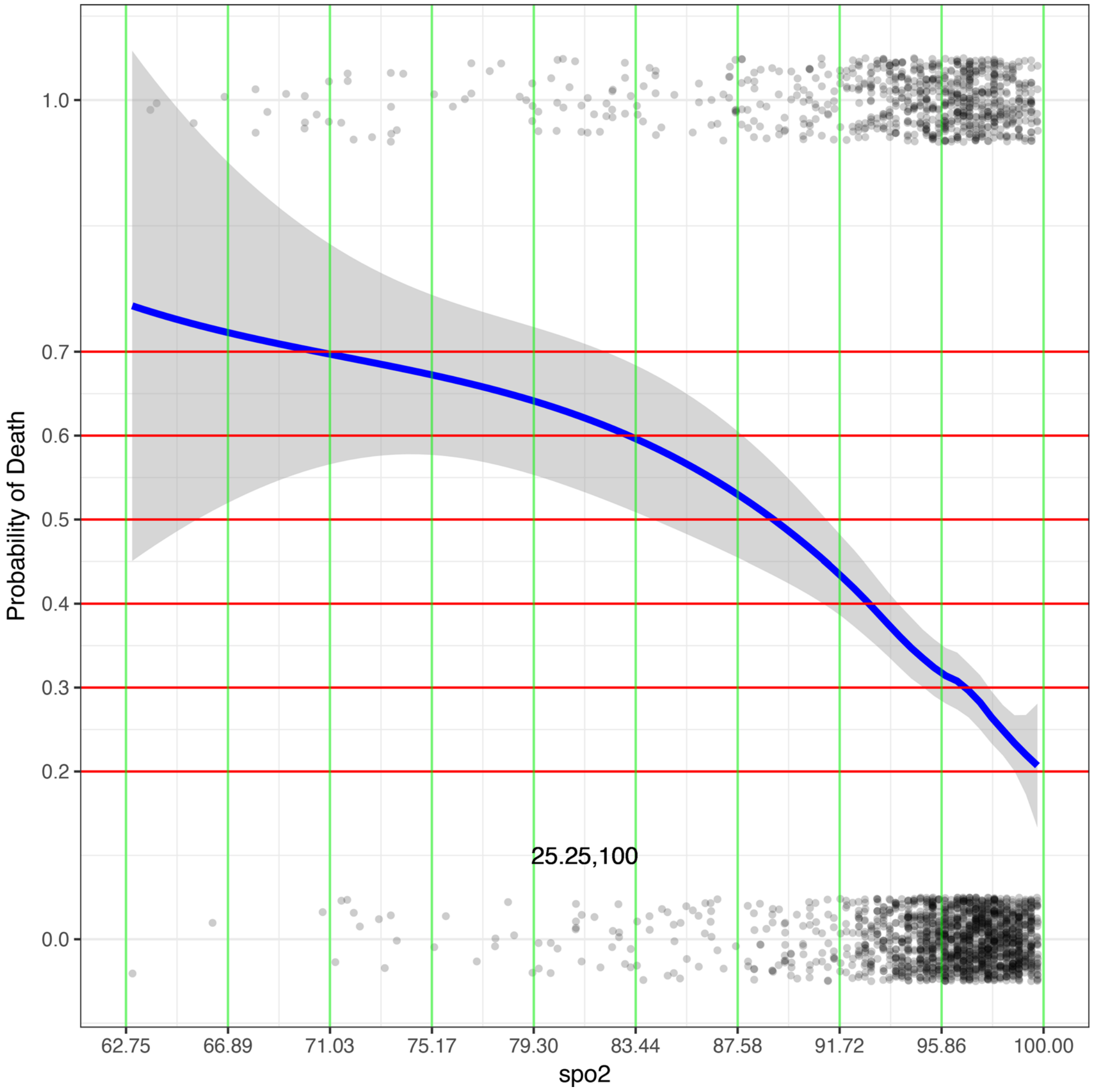

Supplementary Figure 13: Loess smoothing curve showing the association of SPO2 with the risk of death

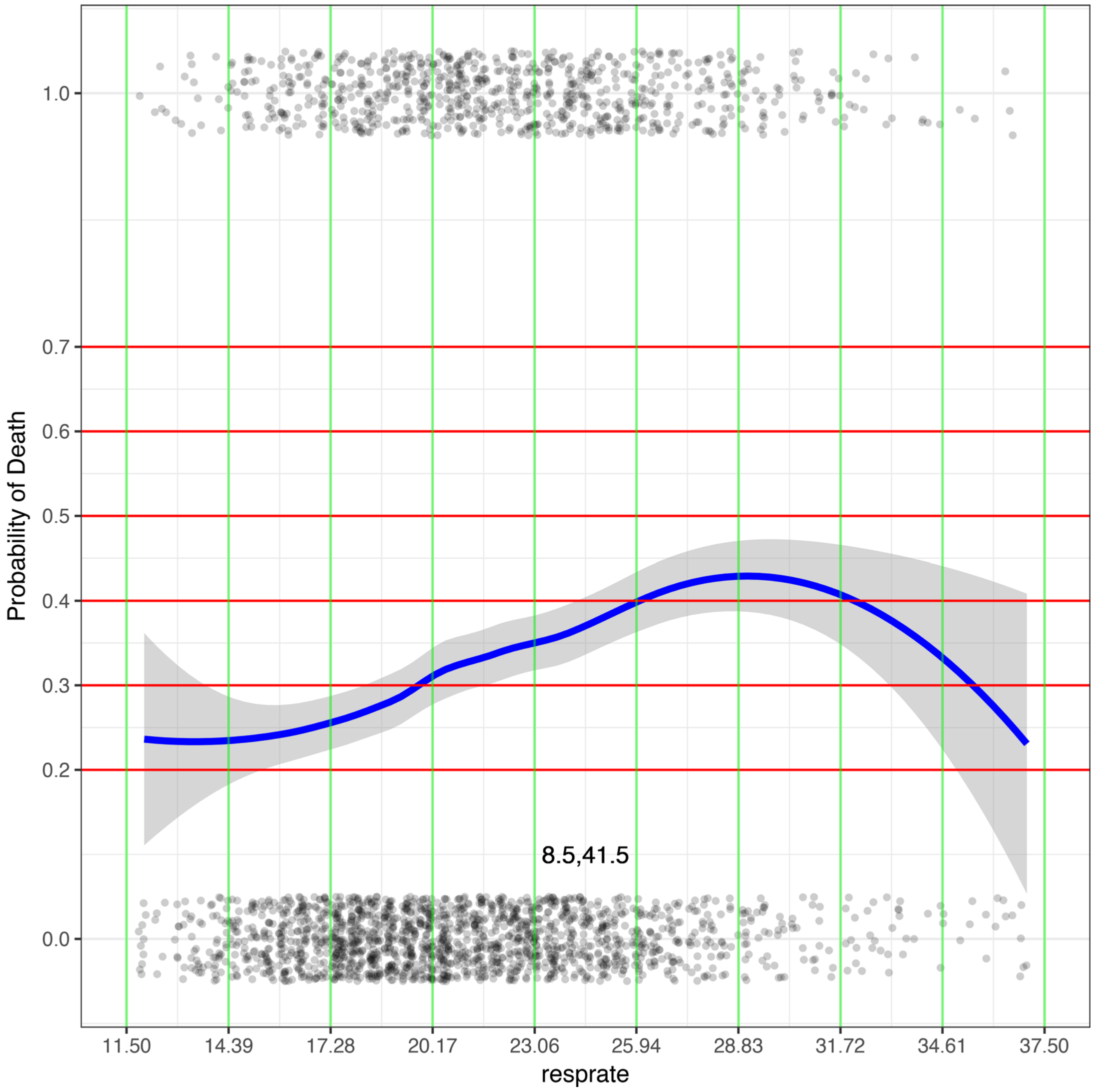

Supplementary Figure 14: Loess smoothing curve showing the association of respiratory rate with the risk of death

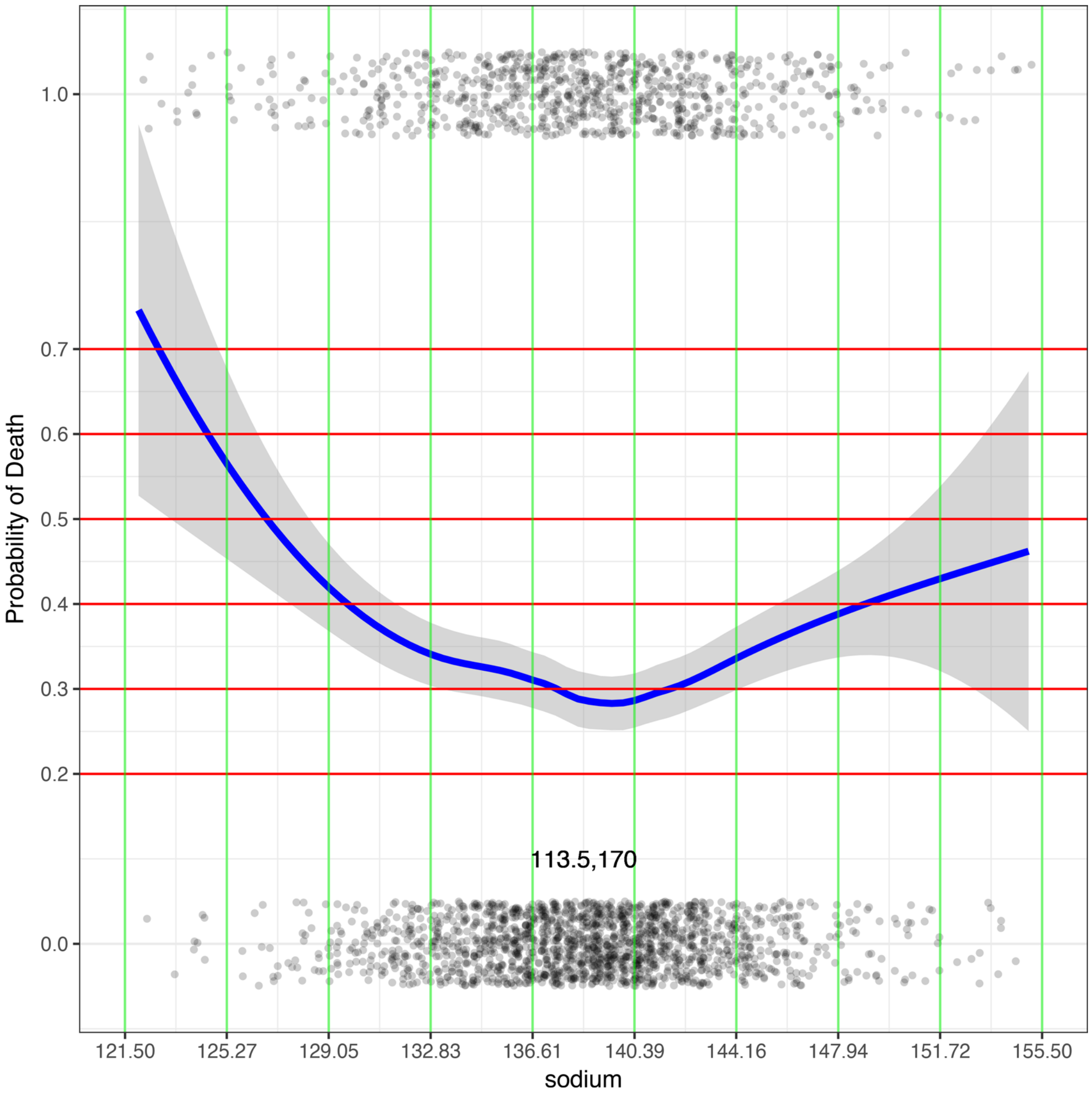

Supplementary Figure 15: Loess smoothing curve showing the association of sodium with the risk of death

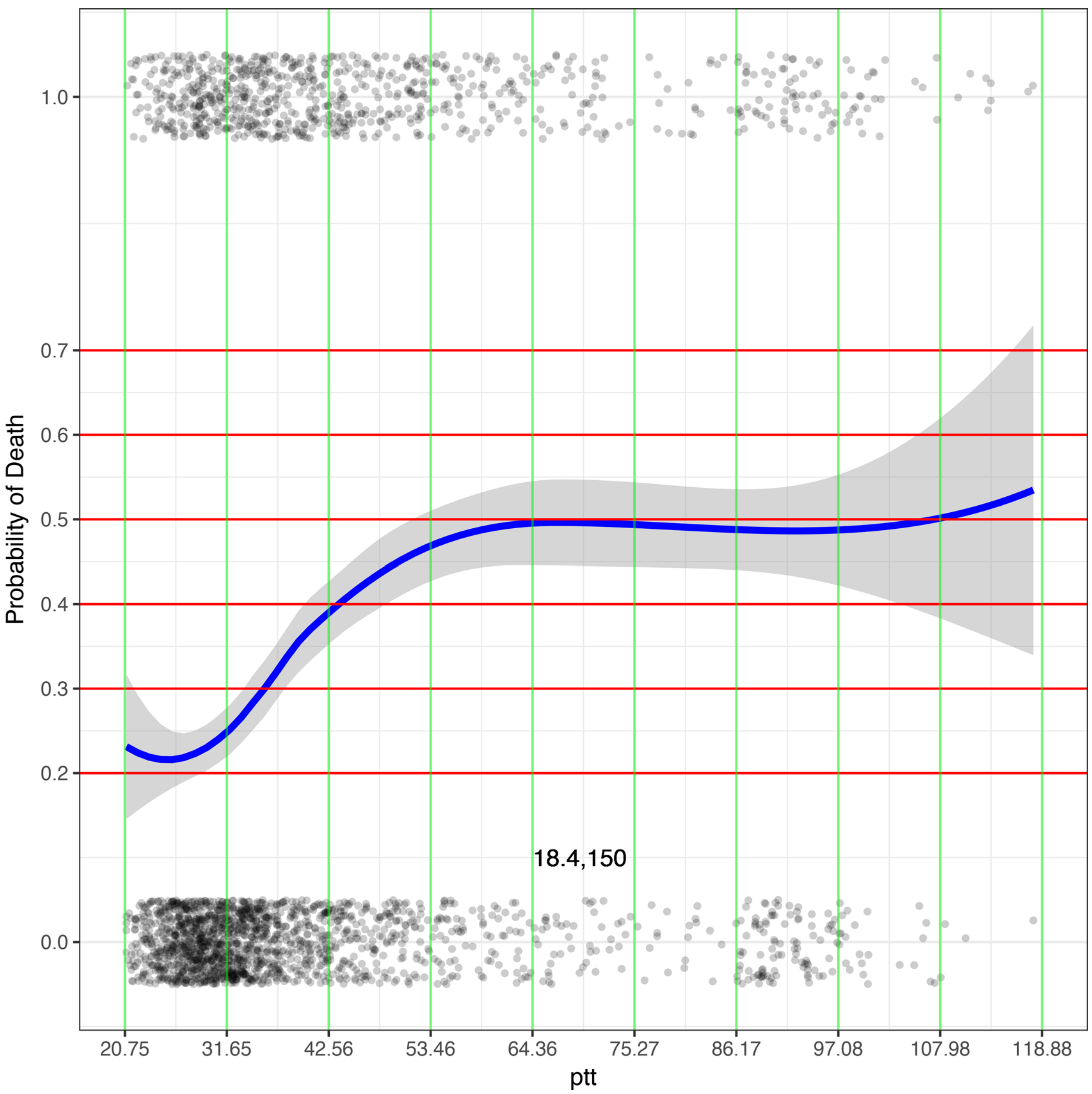

Supplementary Figure 16: Loess smoothing curve showing the association of aPPT with the risk of death

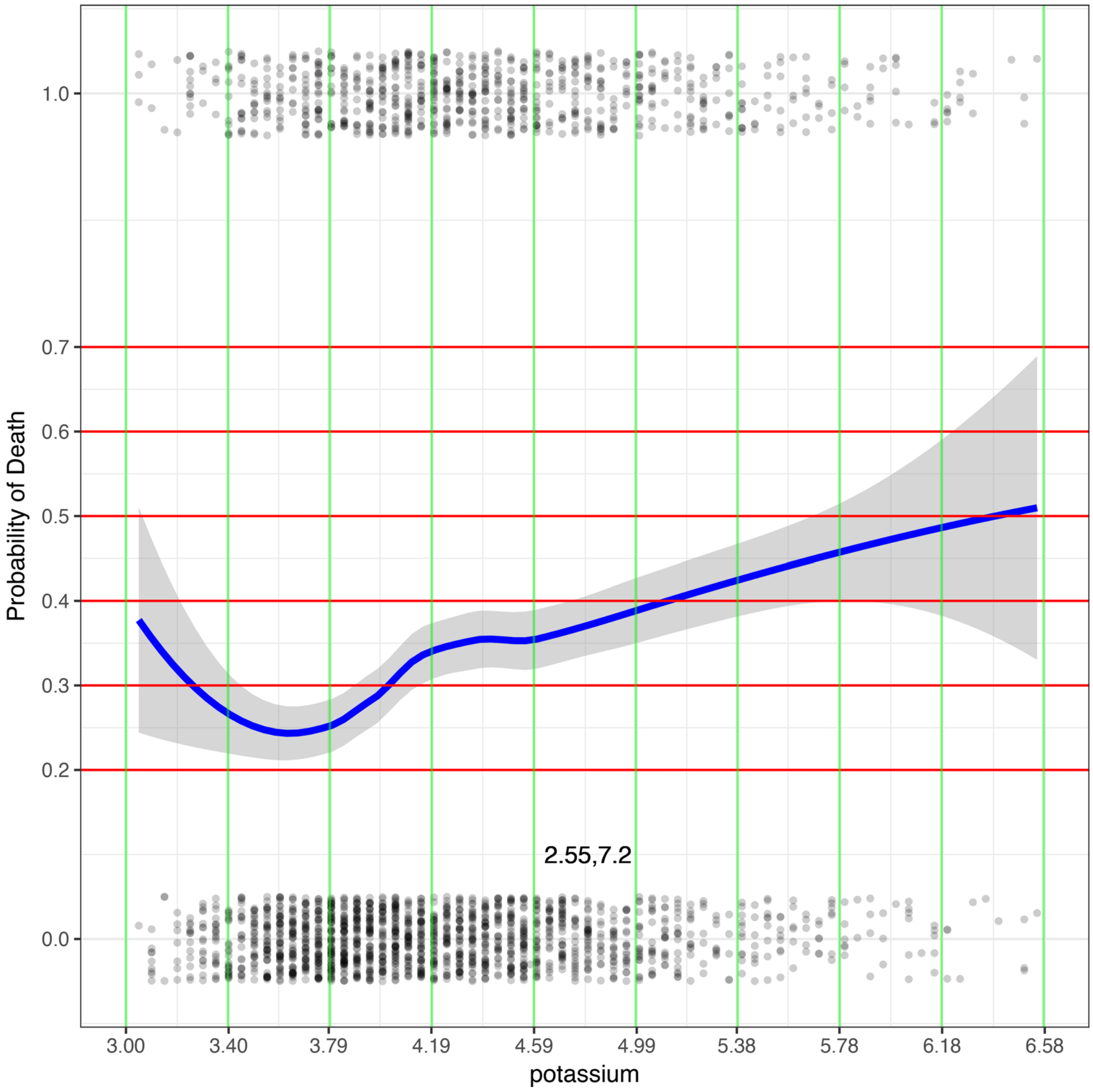

Supplementary Figure 17: Loess smoothing curve showing the association of potassium with the risk of death

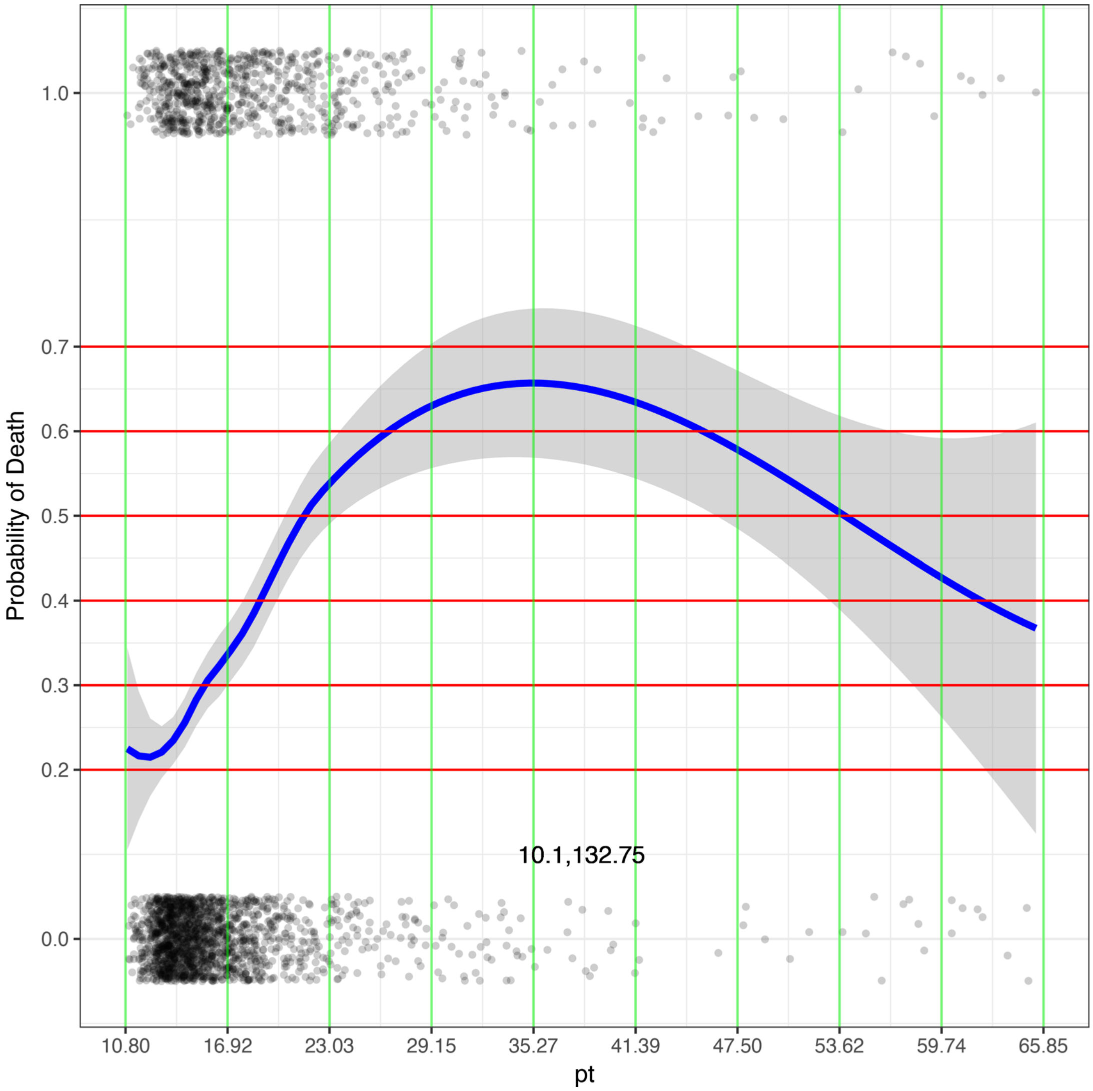

Supplementary Figure 18: Loess smoothing curve showing the association of PT with the risk of death

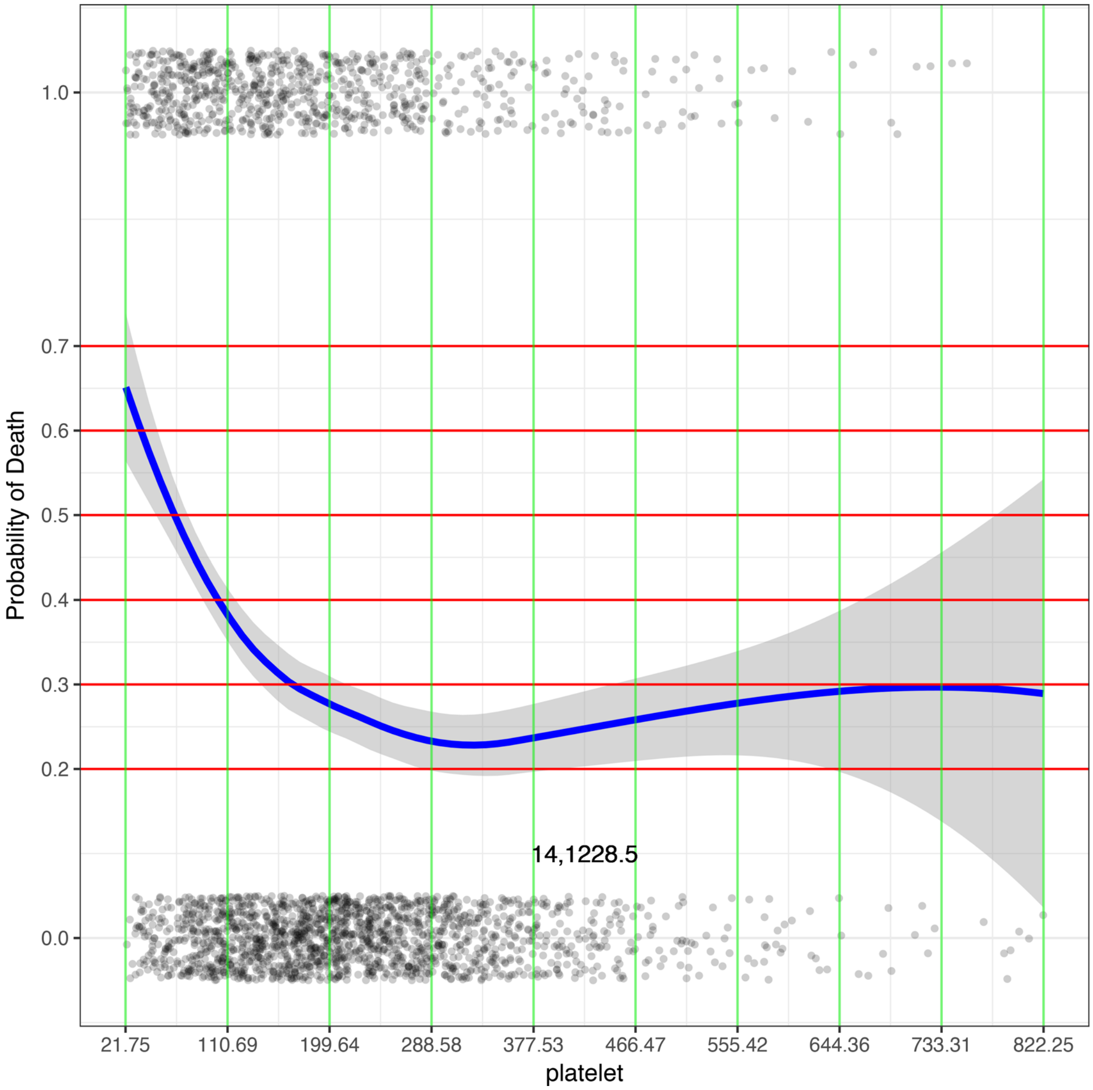

Supplementary Figure 19: Loess smoothing curve showing the association of platelet with the risk of death

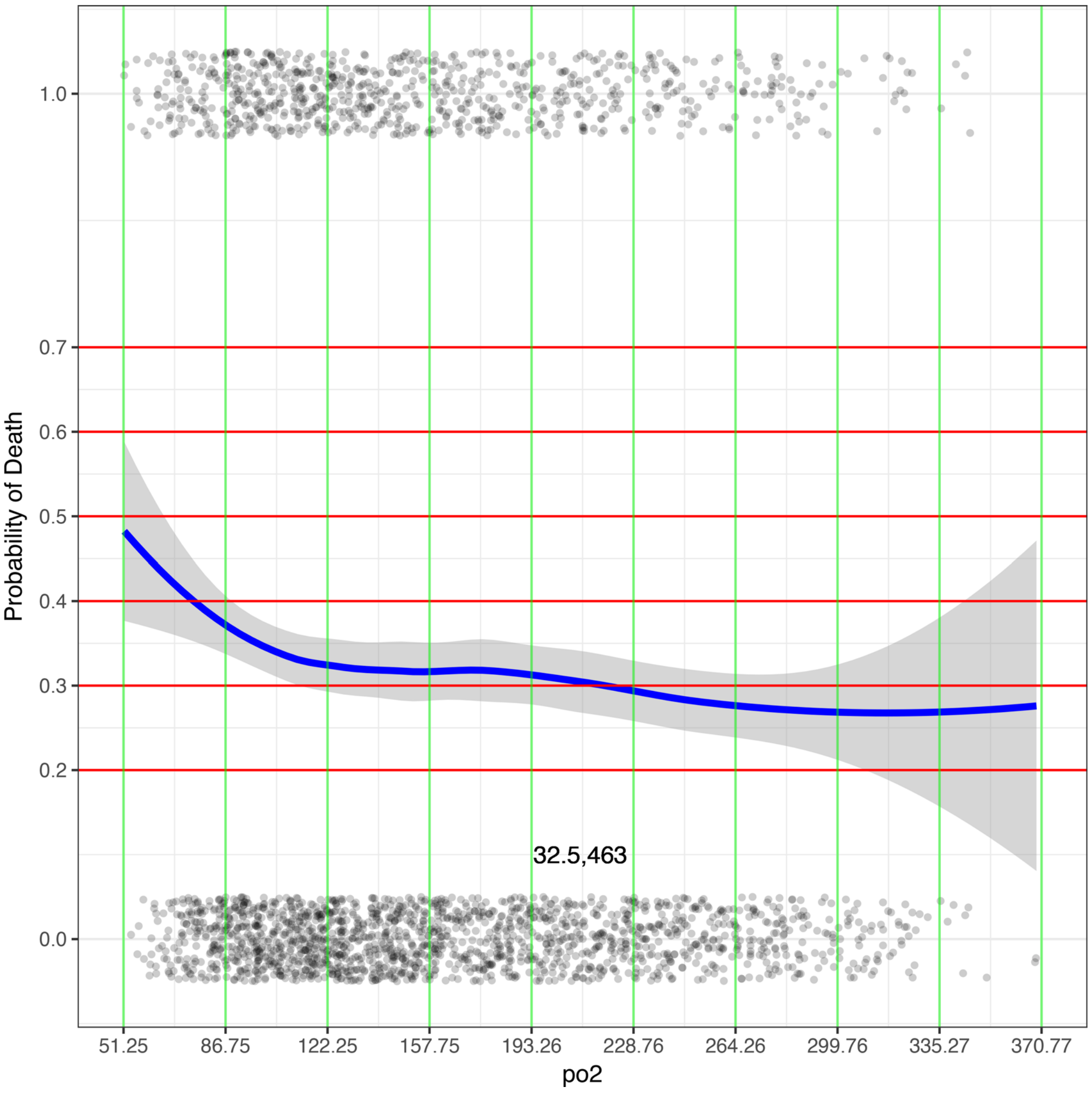

Supplementary Figure 20: Loess smoothing curve showing the association of PaO2 with the risk of death

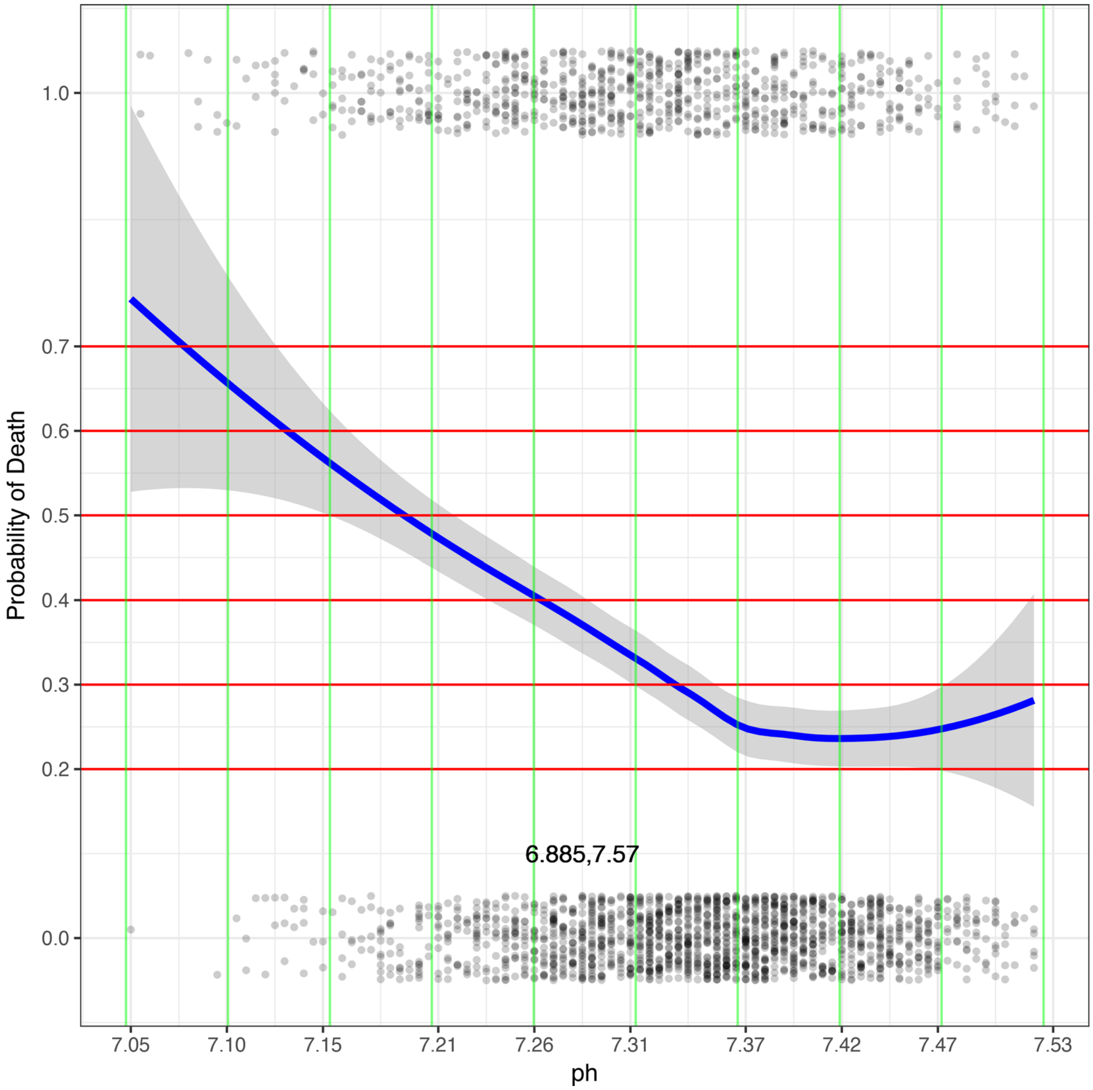

Supplementary Figure 21: Loess smoothing curve showing the association of pH with the risk of death

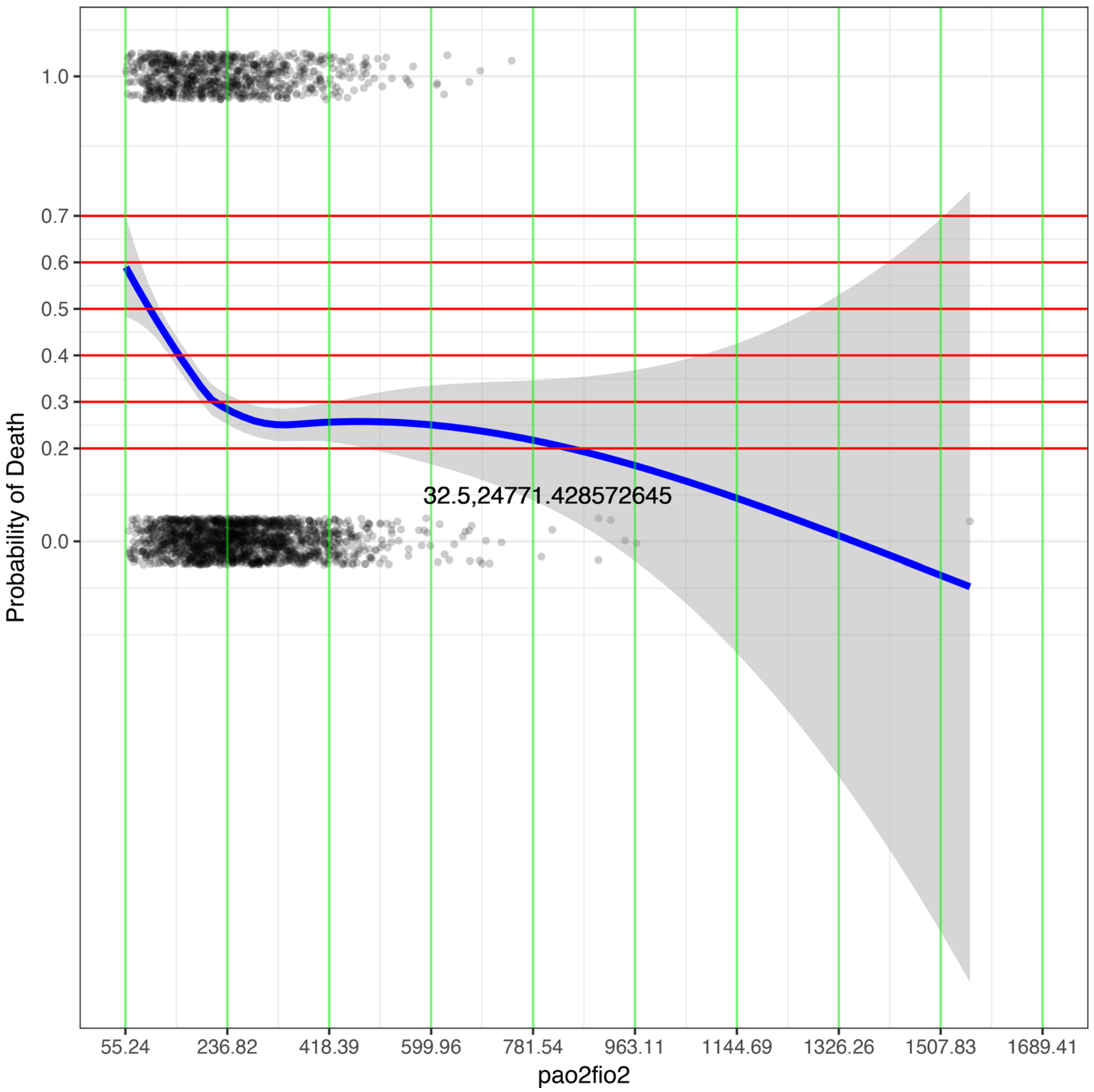

Supplementary Figure 22: Loess smoothing curve showing the association of oxygenation index with the risk of death

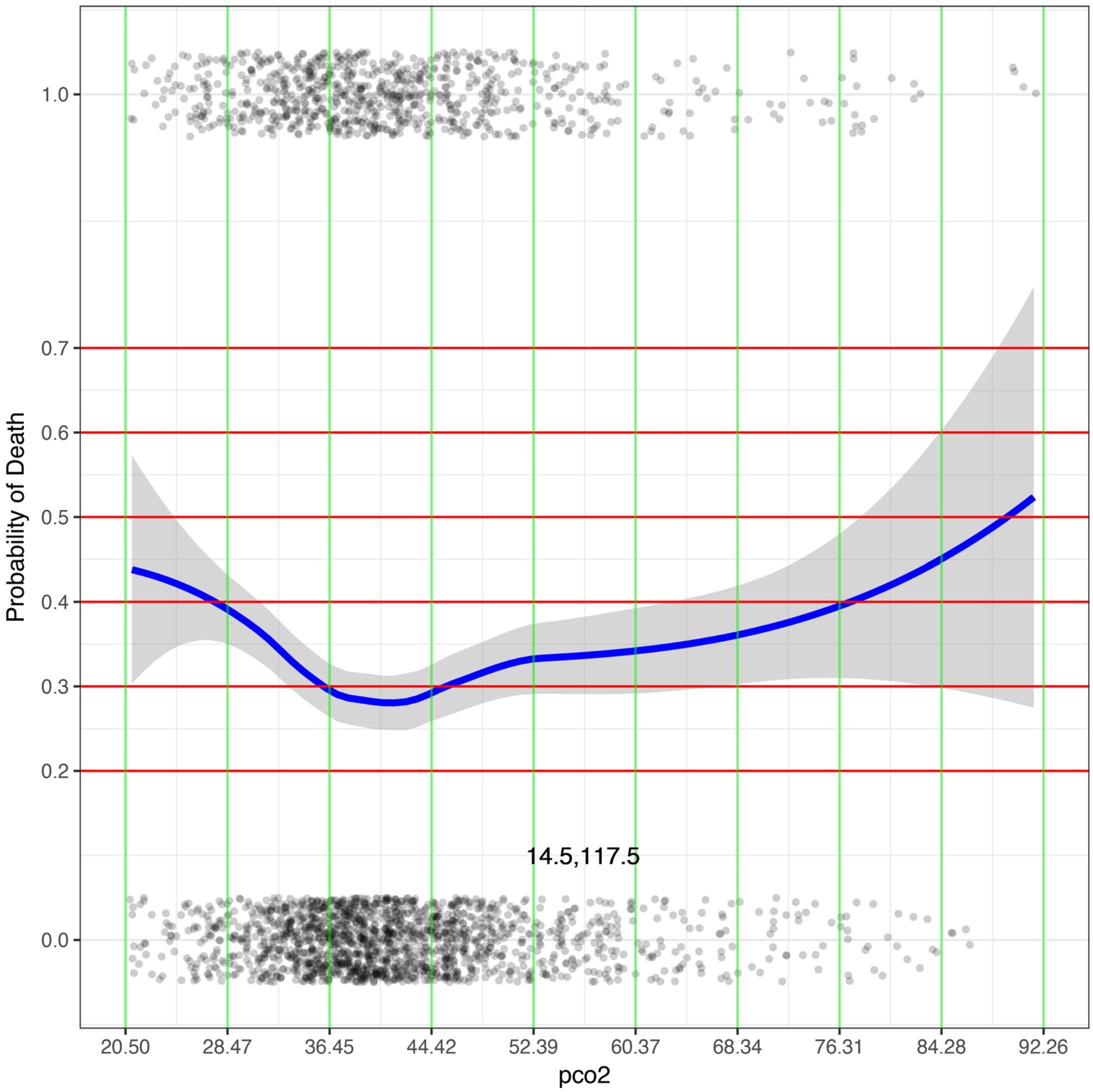

Supplementary Figure 23: Loess smoothing curve showing the association of PaCO2 with the risk of death

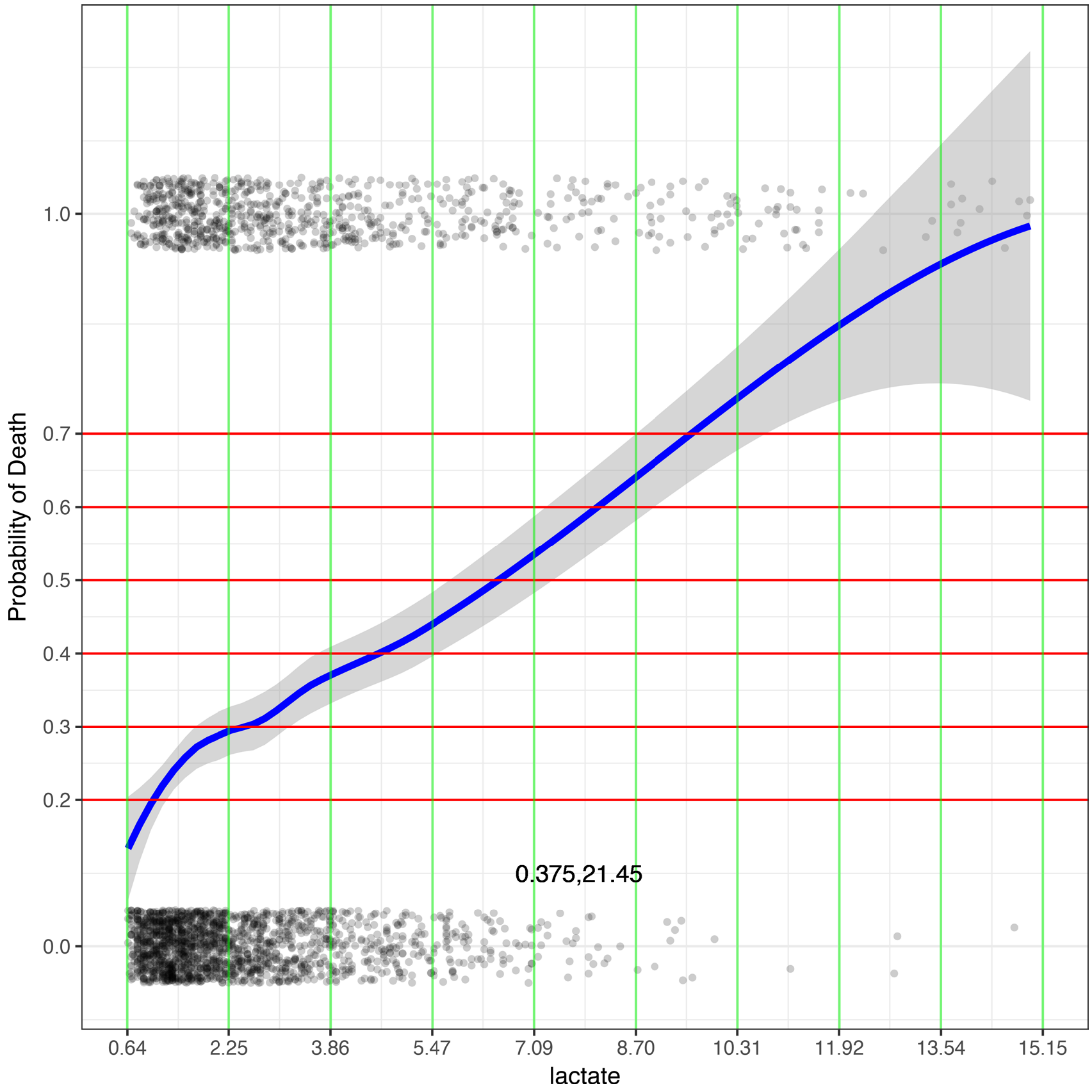

Supplementary Figure 24: Loess smoothing curve showing the association of lactate with the risk of death

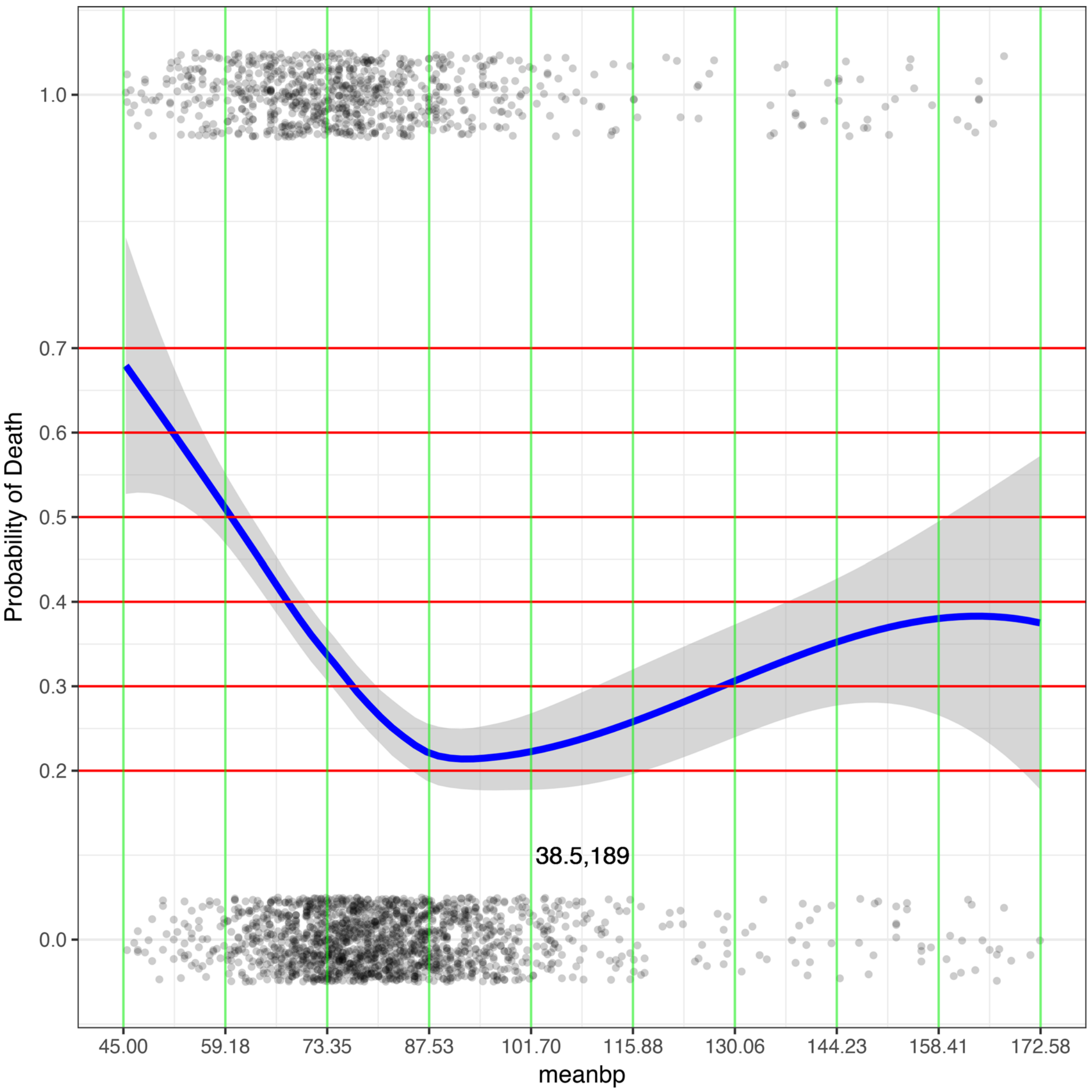

Supplementary Figure 25: Loess smoothing curve showing the association of mean blood pressure with the risk of death

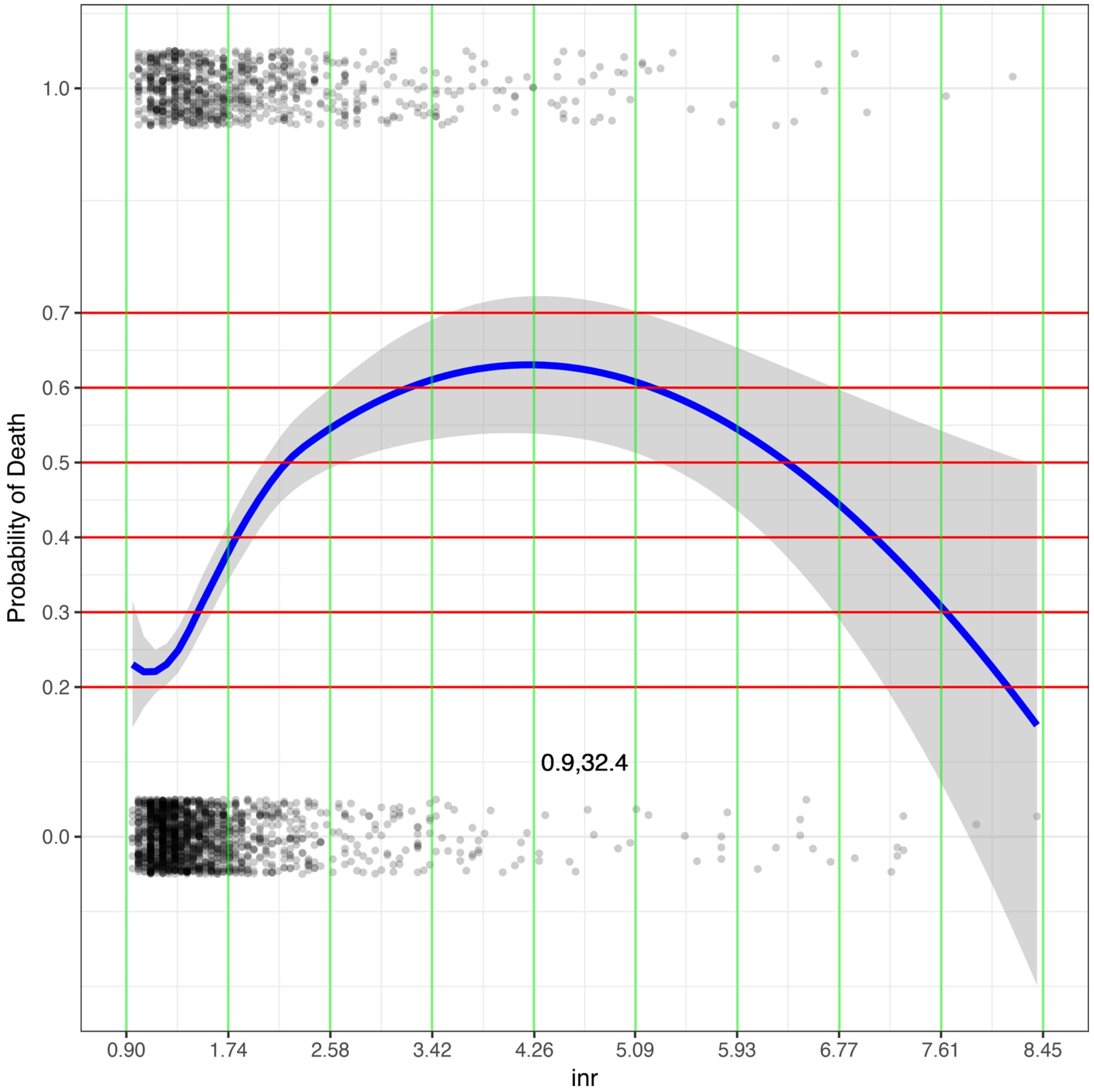

Supplementary Figure 26: Loess smoothing curve showing the association of INR with the risk of death

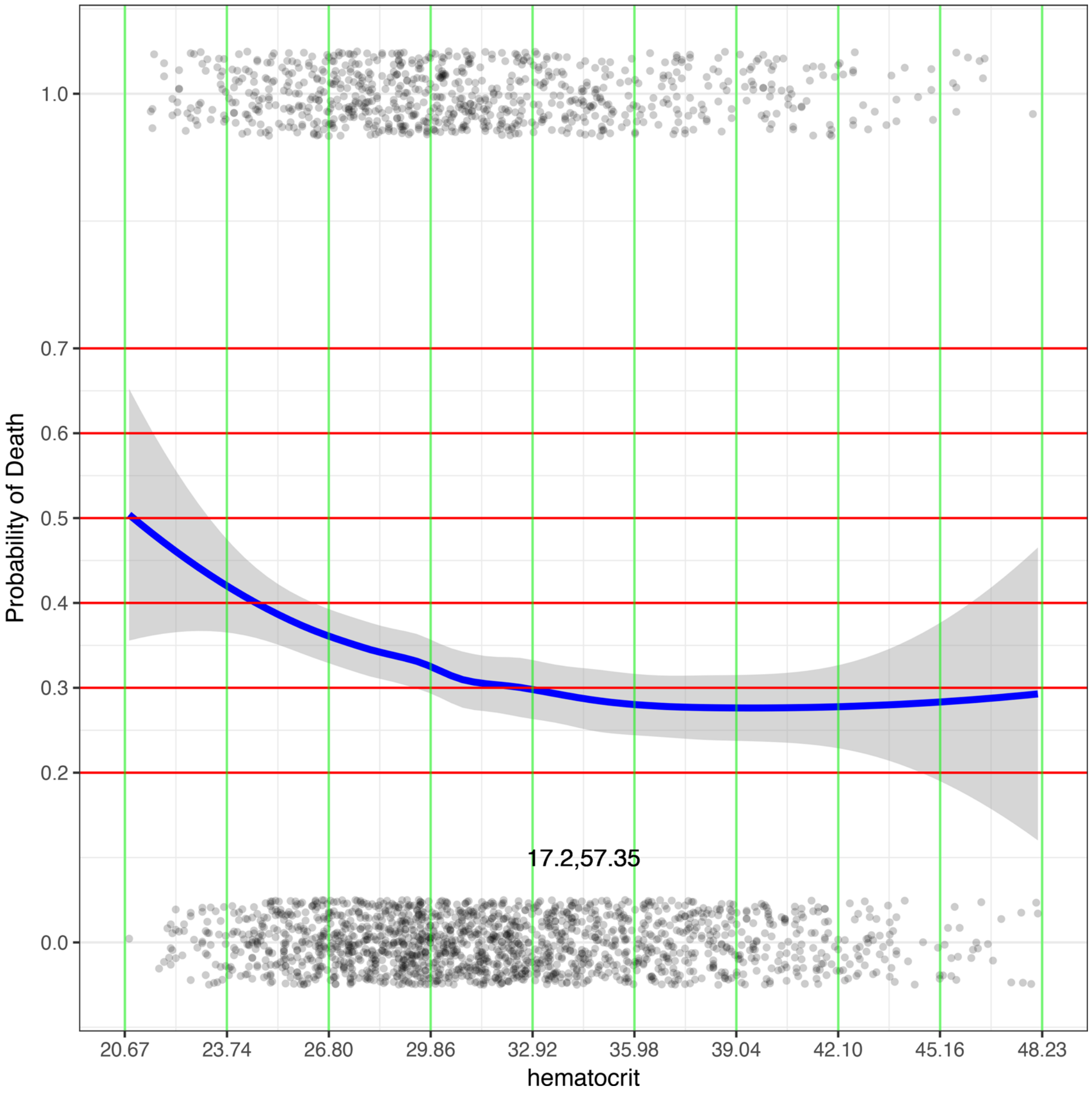

Supplementary Figure 27: Loess smoothing curve showing the association of hematocrit with the risk of death

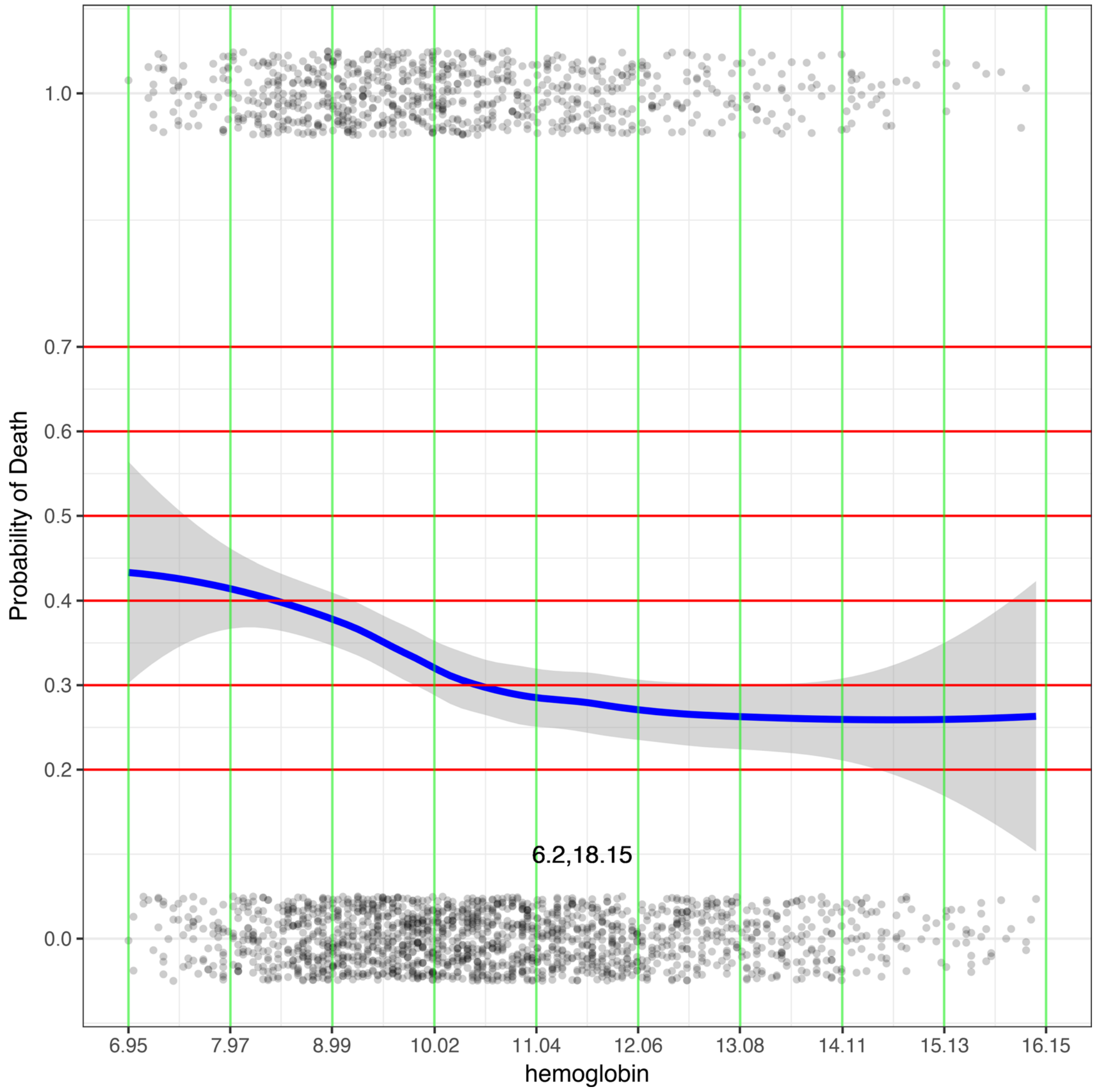

Supplementary Figure 28: Loess smoothing curve showing the association of hemoglobin with the risk of death

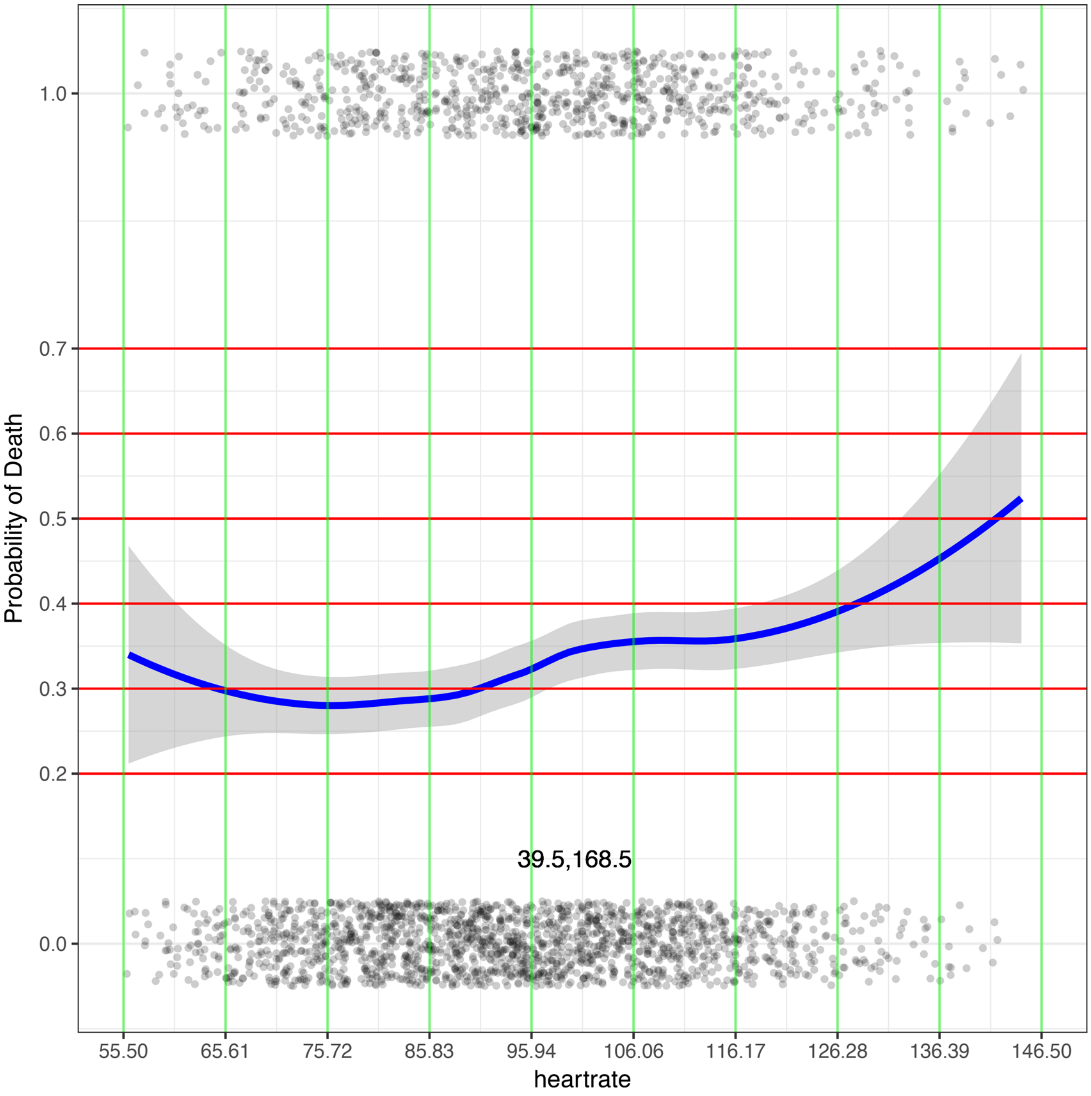

Supplementary Figure 29: Loess smoothing curve showing the association of heart rate with the risk of death

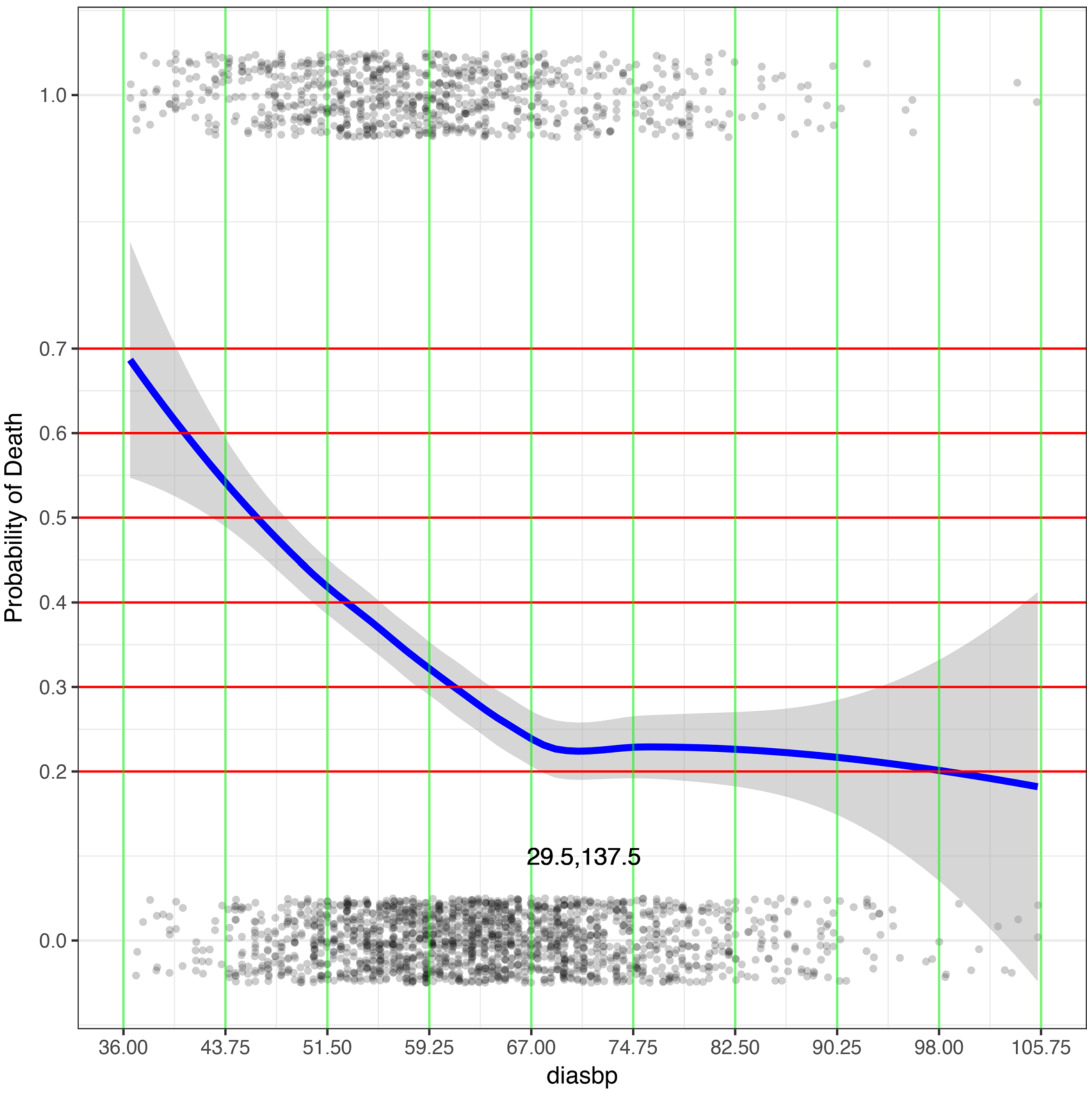

Supplementary Figure 30: Loess smoothing curve showing the association of diastolic blood pressure with the risk of death

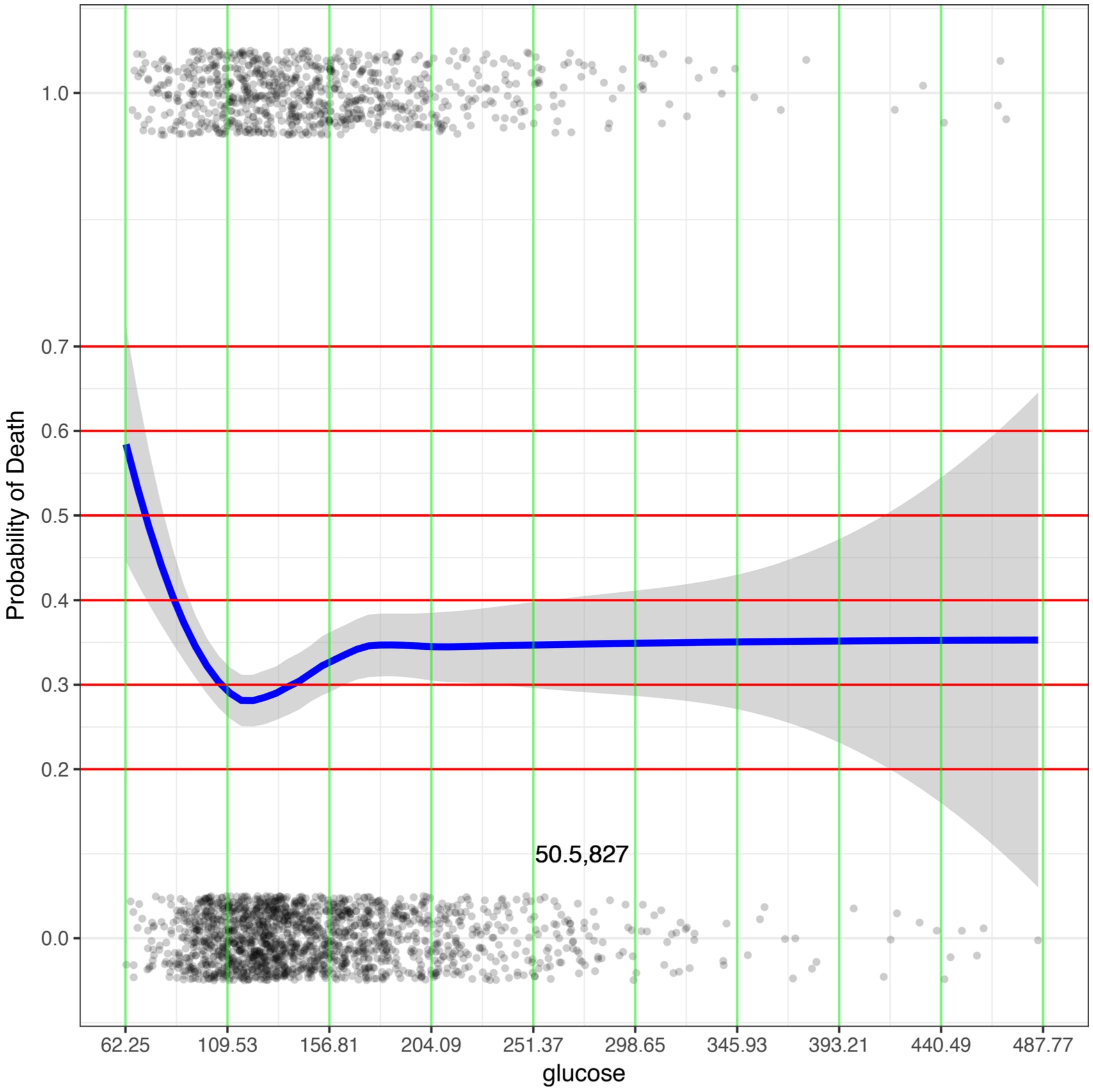

Supplementary Figure 31: Loess smoothing curve showing the association of glucose with the risk of death

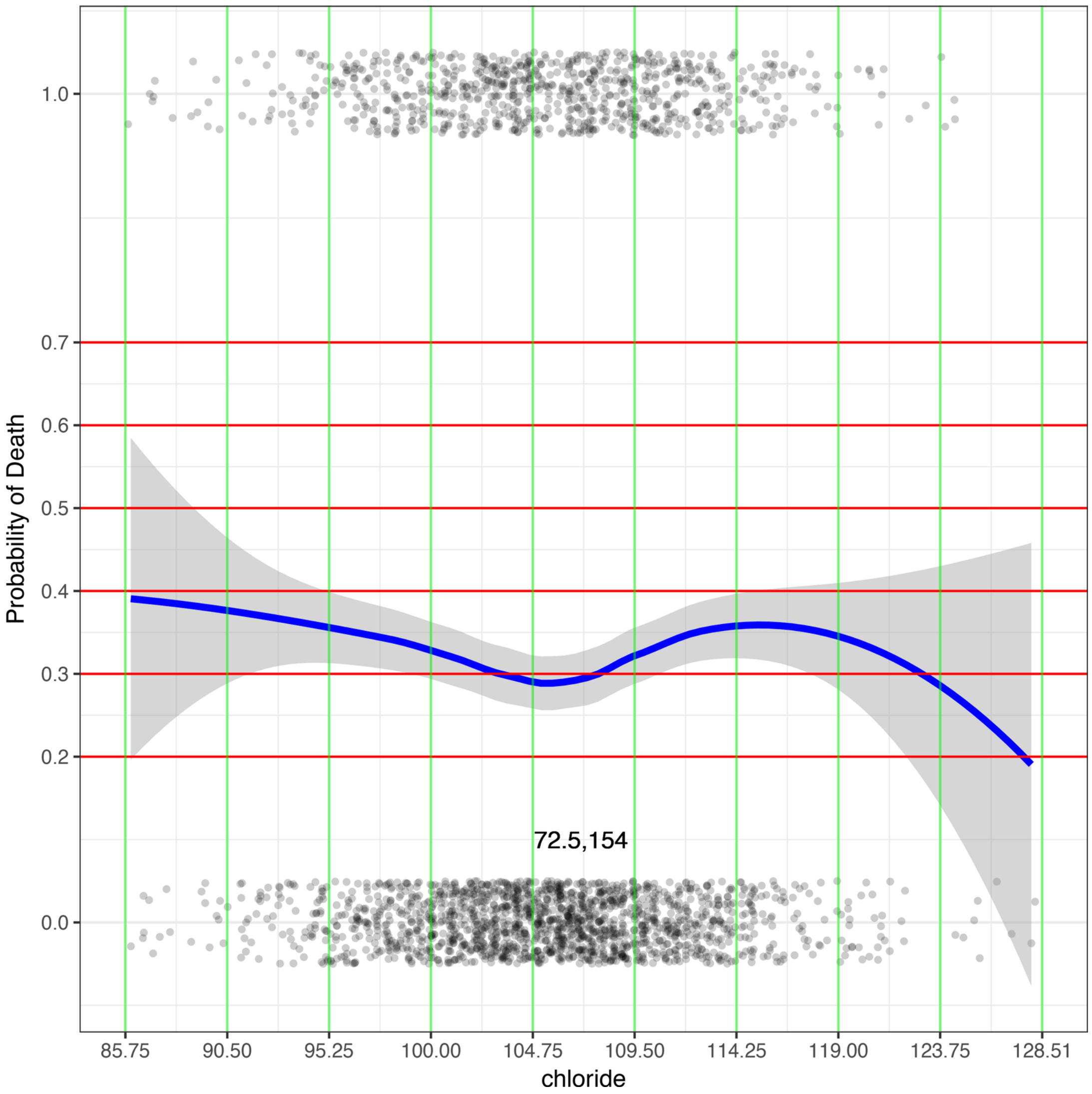

Supplementary Figure 32: Loess smoothing curve showing the association of chloride with the risk of death

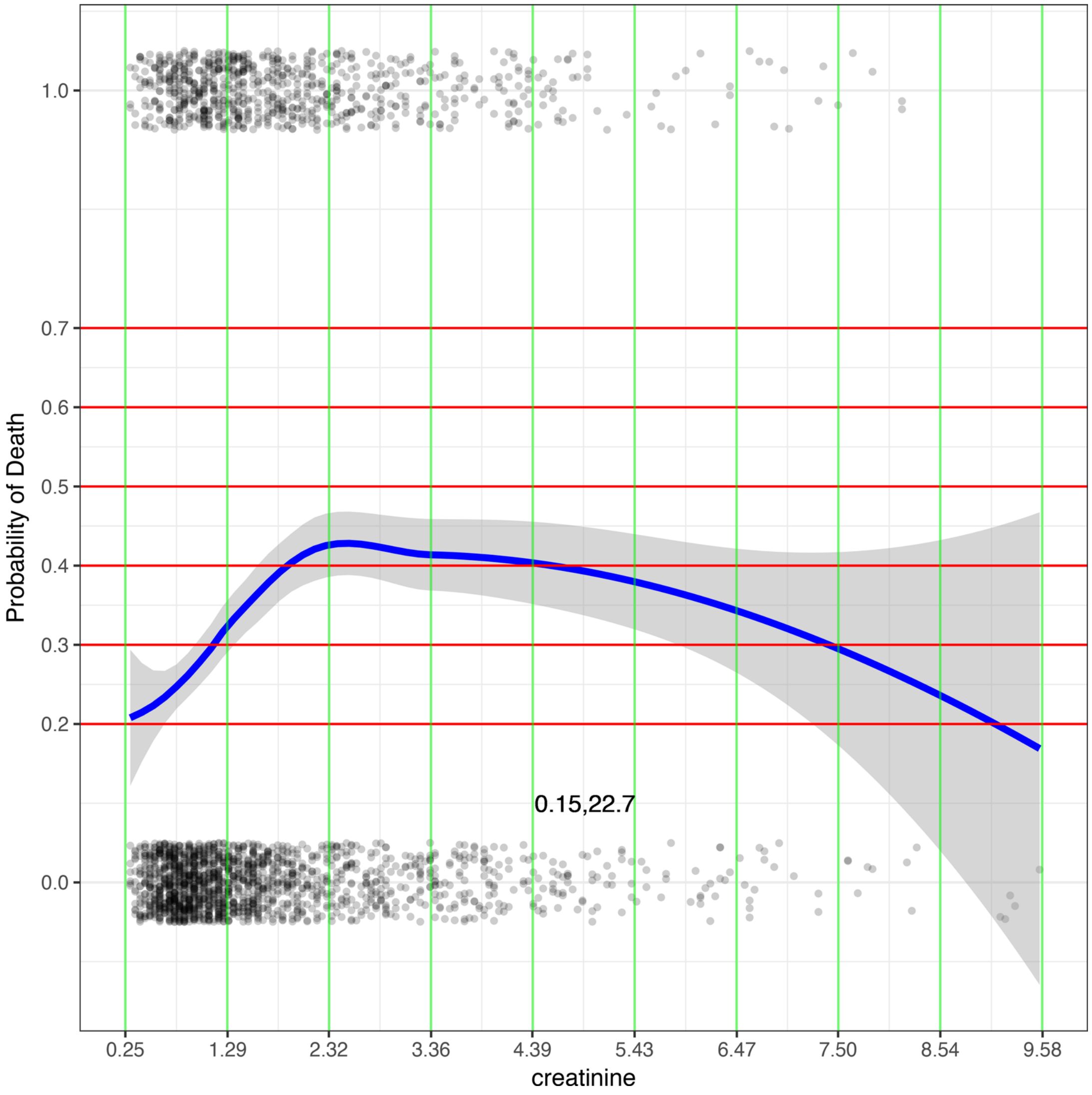

Supplementary Figure 33: Loess smoothing curve showing the association of creatinine with the risk of death

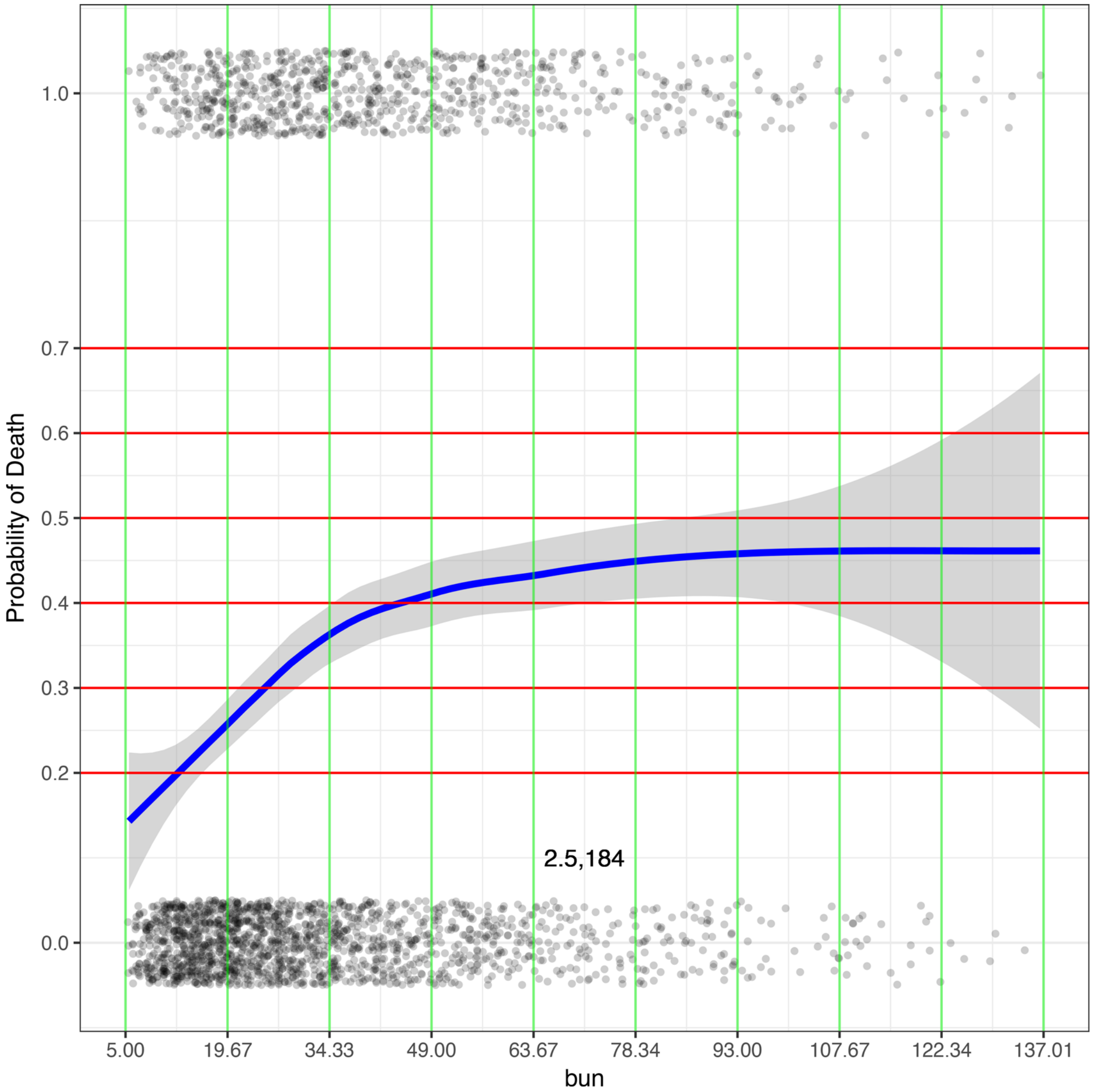

Supplementary Figure 34: Loess smoothing curve showing the association of blood urea nitrogen with the risk of death

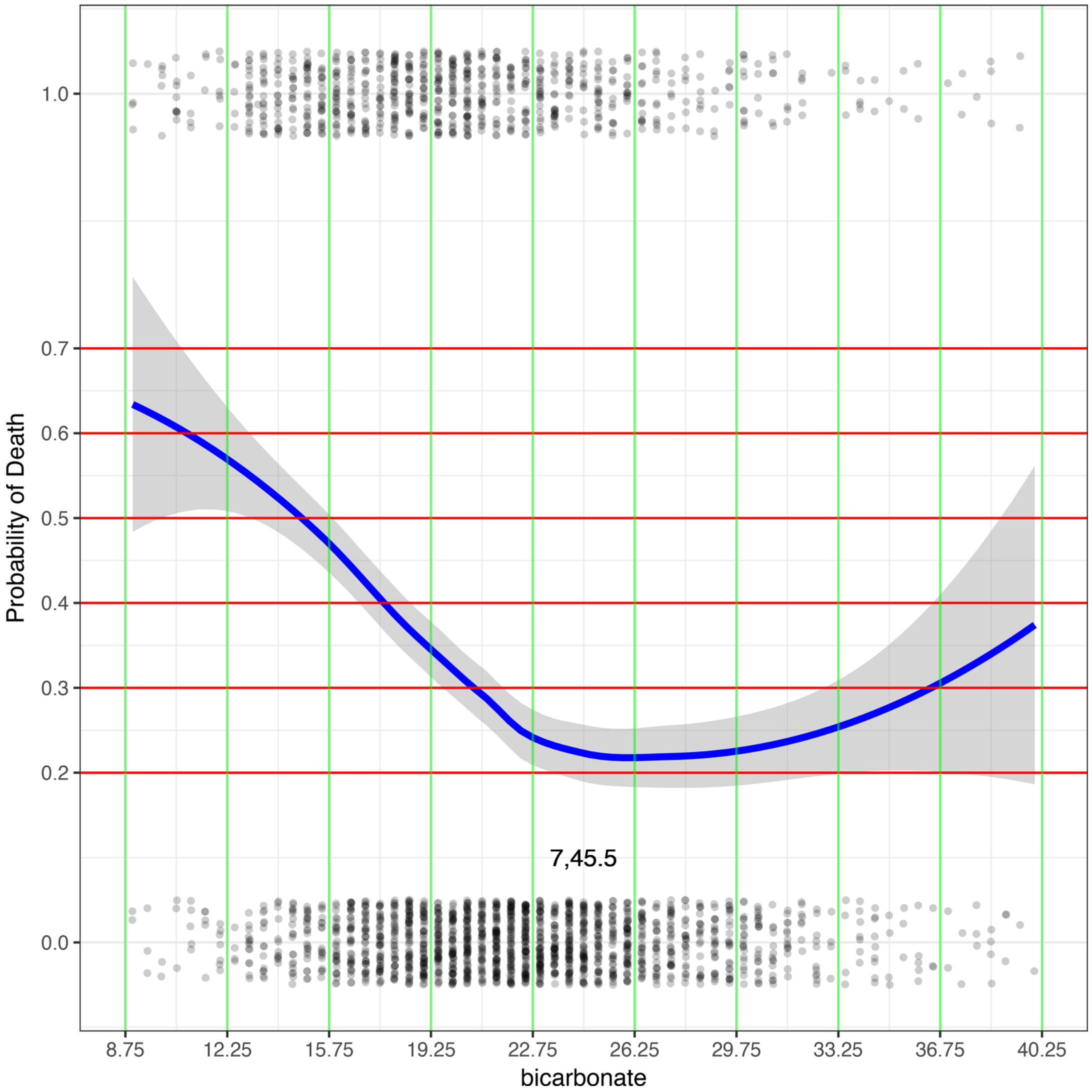

Supplementary Figure 35: Loess smoothing curve showing the association of bicarbonate with the risk of death

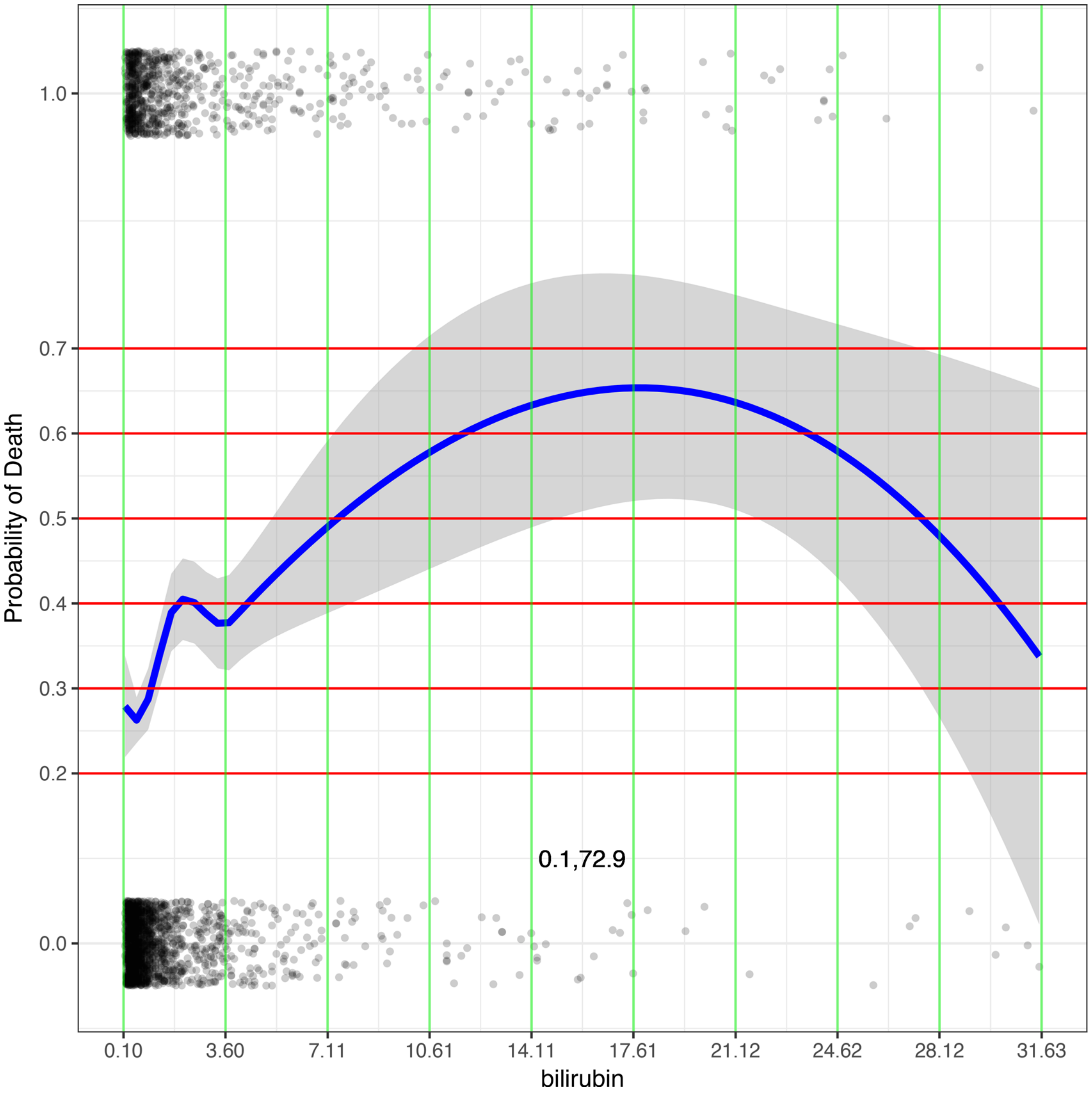

Supplementary Figure 36: Loess smoothing curve showing the association of bilirubin with the risk of death

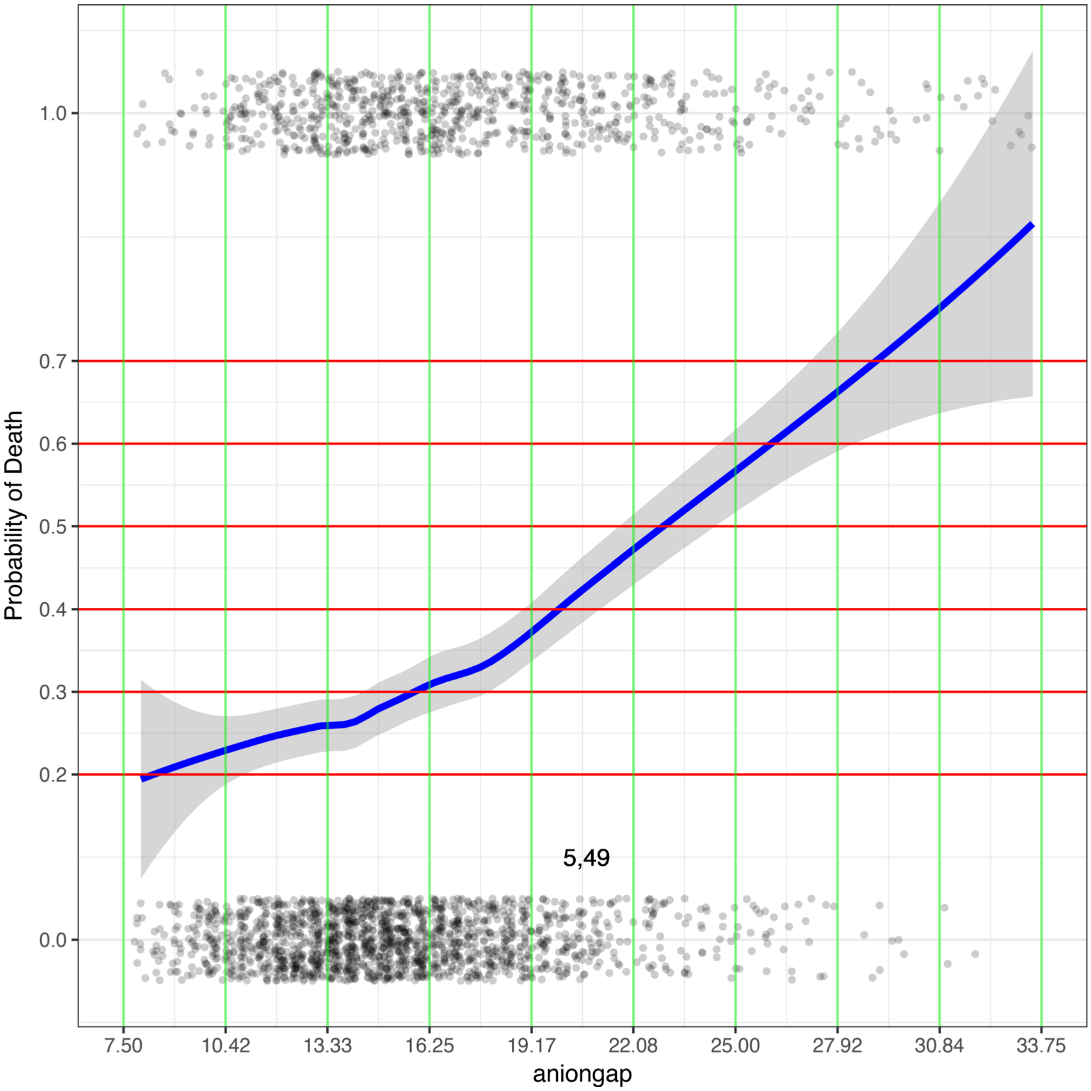

Supplementary Figure 37: Loess smoothing curve showing the association of anion gap with the risk of death

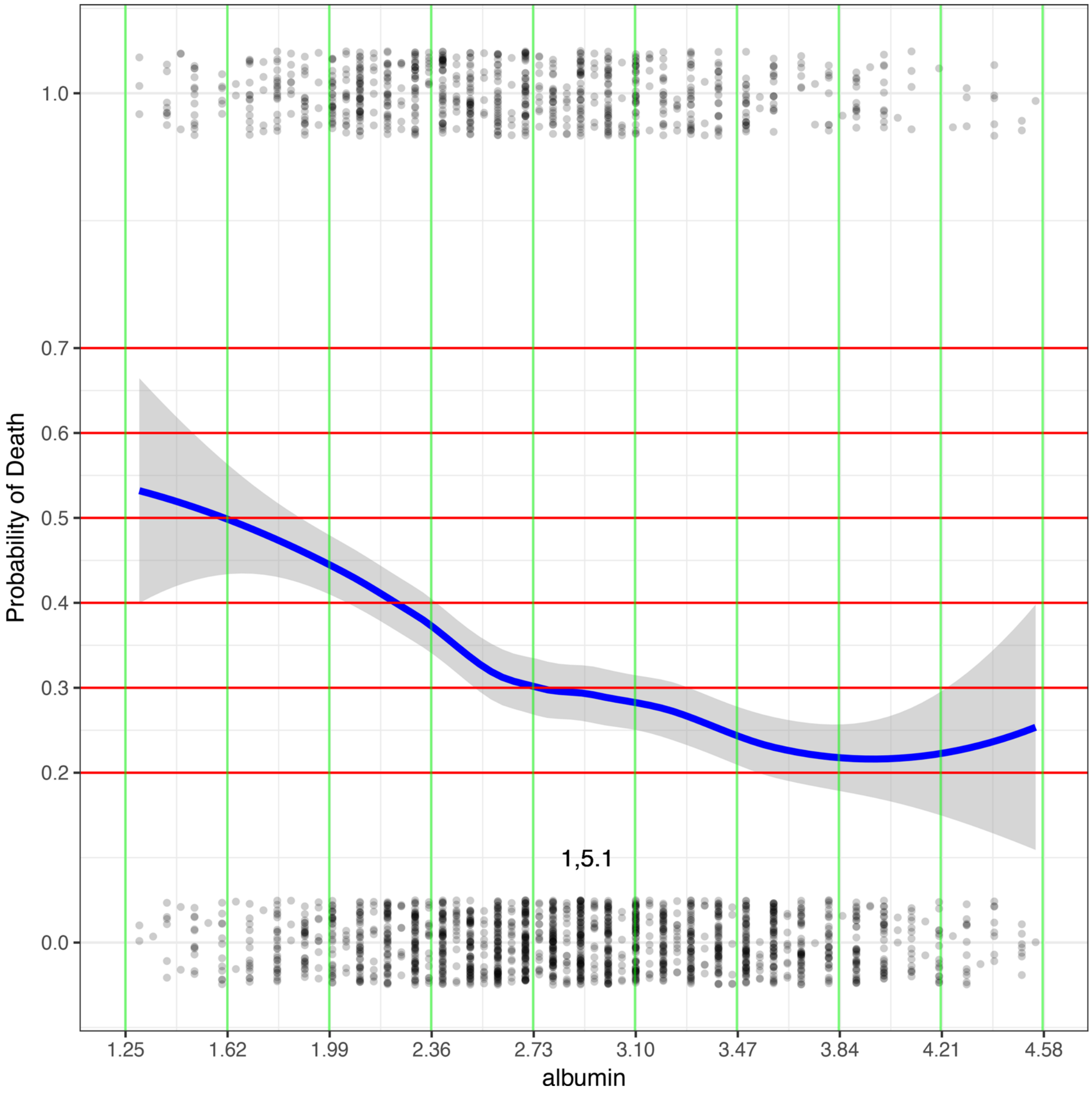

Supplementary Figure 38: Loess smoothing curve showing the association of albumin with the risk of death

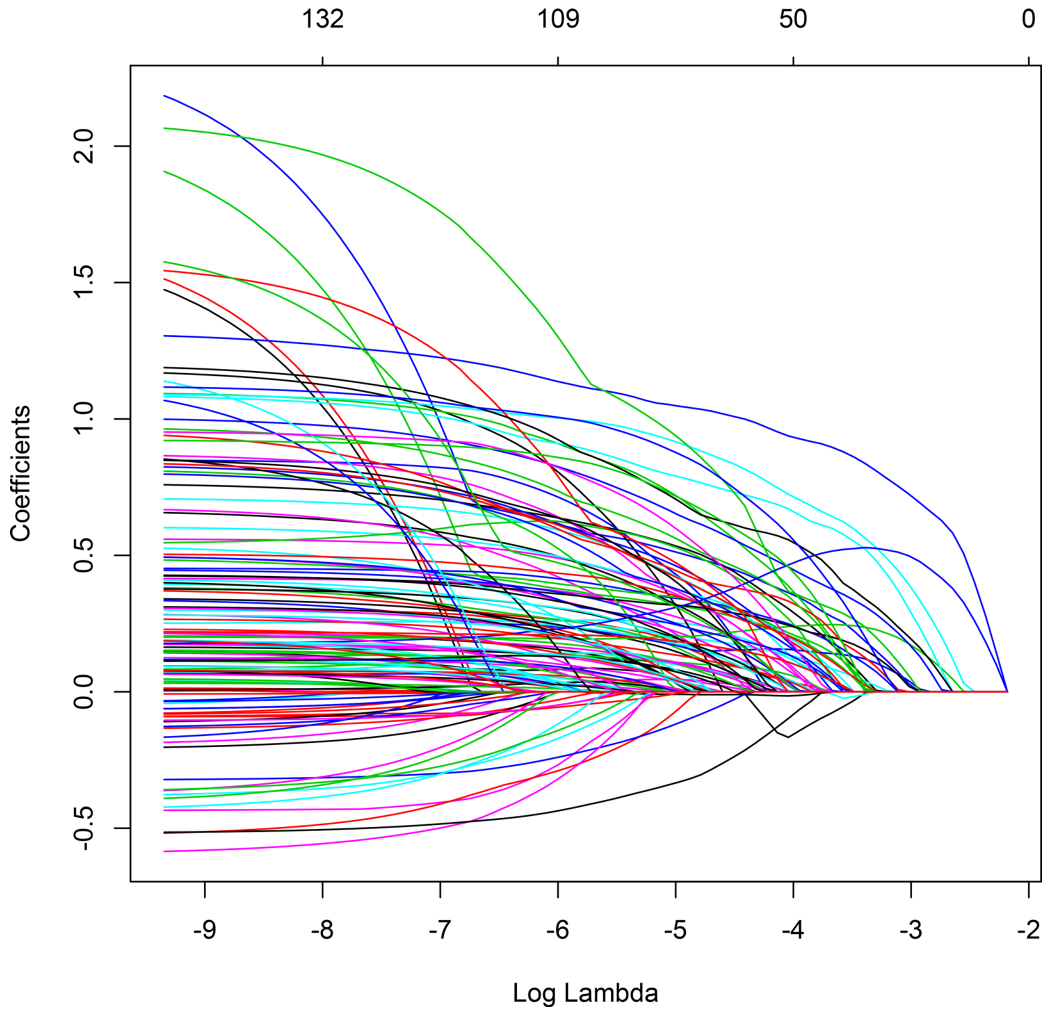

Supplementary Figure 39: Coefficient profile with changing values of Lambda. Lambda is a tuning parameter controlling the overall strength of the penalty. By using lasso method, some less important coefficients tend to be discarded.

Supplementary Table 1: Comparisons between survivors and non-survivors for continuous variables

	Mean.total	Sd.total	mean.die	sd.die	mean.alive	sd.alive	*p*
aado2_calc	252.98	135.44	289.62	141.16	235.04	128.83	0.000
albumin	2.83	0.67	2.66	0.68	2.91	0.65	0.000
aniongap	16.03	4.66	17.63	5.60	15.25	3.90	0.000
bicarbonate	21.84	5.70	20.38	5.94	22.55	5.43	0.000
bilirubin	2.33	4.99	3.35	5.98	1.83	4.34	0.000
bun	37.64	26.50	44.47	28.05	34.30	25.05	0.000
chloride	105.42	6.89	105.21	7.04	105.53	6.82	0.232
creatinine	1.86	1.65	2.06	1.55	1.77	1.68	0.000
diasbp	62.44	12.23	59.58	12.71	63.85	11.75	0.000
glucose	155.07	63.09	156.02	64.83	154.61	62.22	0.556
heartrate	94.88	18.12	96.58	18.86	94.04	17.70	0.000
hematocrit	31.73	5.49	31.15	5.60	32.02	5.41	0.000
hemoglobin	10.56	1.87	10.30	1.88	10.69	1.85	0.000
inr	1.77	1.38	2.05	1.58	1.64	1.25	0.000
lactate	2.98	2.48	4.03	3.34	2.46	1.70	0.000
meanbp	81.93	20.39	78.84	21.58	83.45	19.61	0.000
pao2fio2	302.05	792.75	252.32	497.99	326.40	901.78	0.003
pco2	42.18	11.99	41.65	12.88	42.45	11.52	0.087
ph	7.34	0.09	7.31	0.10	7.35	0.08	0.000
platelet	217.51	138.12	192.78	138.56	229.63	136.31	0.000
po2	164.41	69.61	160.24	71.06	166.45	68.82	0.019
potassium	4.29	0.68	4.40	0.71	4.23	0.66	0.000
pt	17.85	8.64	19.88	10.35	16.86	7.47	0.000
ptt	43.00	20.99	48.11	22.94	40.50	19.49	0.000
resprate	21.44	4.82	22.22	4.86	21.05	4.76	0.000
sodium	138.64	5.49	138.32	5.96	138.80	5.23	0.025
spo2	95.10	5.66	93.50	7.53	95.88	4.26	0.000
sysbp	116.23	16.83	110.67	16.91	118.95	16.12	0.000
tempc	36.89	0.89	36.67	1.01	37.00	0.80	0.000
totalco2	23.30	6.37	21.76	6.62	24.05	6.11	0.000
wbc	14.53	12.77	15.67	18.69	13.97	8.43	0.005
age	73.52	51.37	80.11	57.75	70.29	47.61	0.000
mingcs	13.21	3.38	13.00	3.73	13.31	3.19	0.022
urineoutput	1529.21	1534.47	1077.90	1134.26	1750.25	1652.23	0.000
rate_dopamine	1.81	7.97	2.67	7.01	1.38	8.37	0.000
rate_epinephrine	0.00	0.05	0.01	0.08	0.00	0.02	0.012
rate_norepinephrine	0.16	1.17	0.20	0.42	0.14	1.39	0.049
rate_dobutamine	0.20	1.38	0.30	1.80	0.15	1.12	0.009

Supplementary Table 2: Comparisons between survivors and non-survivors for categorical variables. See Supplementary_Table_2.

Supplementary Table 3: The LASSO score. See Supplementary_Table_3.
