# Supplementary material for: Development of a novel score for the prediction of hospital mortality in patients with severe sepsis: the use of electronic healthcare records with LASSO regression
Source: Oncotarget. 2017 May 15;8(30):49637–45. doi: 10.18632/oncotarget.17870 (PMC5564794; doi:10.18632/oncotarget.17870)
Supplement: Supplementary file 2 [file oncotarget-08-49637-s002.docx]

# Table 2 Comparisons between survivors and non-survivors for categorical variables

|  | No.tot | per.tot | No.die | per.die | No.alive | per.alive | p |
| --- | --- | --- | --- | --- | --- | --- | --- |
| gender_F | 1346 | 0.42 | 423 | 0.40 | 923 | 0.43 | 0.148 |
| gender_M | 1860 | 0.58 | 631 | 0.60 | 1229 | 0.57 | 0.148 |
| gender_F1 | 1346 | 0.42 | 423 | 0.40 | 923 | 0.43 | 0.148 |
| gender_M1 | 1860 | 0.58 | 631 | 0.60 | 1229 | 0.57 | 0.148 |
| ethnicity_ASIAN | 82 | 0.03 | 24 | 0.02 | 58 | 0.03 | 0.000 |
| ethnicity_BLACK | 242 | 0.08 | 70 | 0.07 | 172 | 0.08 | 0.000 |
| ethnicity_HISPANIC | 102 | 0.03 | 30 | 0.03 | 72 | 0.03 | 0.000 |
| ethnicity_UNKNOWN | 442 | 0.14 | 198 | 0.19 | 244 | 0.11 | 0.000 |
| ethnicity_WHITE | 2338 | 0.73 | 732 | 0.69 | 1606 | 0.75 | 0.000 |
| admission_type_ELECTIVE | 159 | 0.05 | 39 | 0.04 | 120 | 0.06 | 0.066 |
| admission_type_EMERGENCY | 2958 | 0.92 | 987 | 0.94 | 1971 | 0.92 | 0.066 |
| admission_type_URGENT | 89 | 0.03 | 28 | 0.03 | 61 | 0.03 | 0.066 |
| hospital_expire_flag_0 | 2152 | 0.67 | 0 | 0.00 | 2152 | 1.00 | 0.000 |
| hospital_expire_flag_1 | 1054 | 0.33 | 1054 | 1.00 | 0 | 0.00 | 0.000 |
| electivesurgery_0 | 3069 | 0.96 | 1021 | 0.97 | 2048 | 0.95 | 0.032 |
| electivesurgery_1 | 137 | 0.04 | 33 | 0.03 | 104 | 0.05 | 0.032 |
| rrt_0 | 2934 | 0.92 | 939 | 0.89 | 1995 | 0.93 | 0.001 |
| rrt_1 | 272 | 0.08 | 115 | 0.11 | 157 | 0.07 | 0.001 |
| mechvent_0 | 226 | 0.07 | 52 | 0.05 | 174 | 0.08 | 0.001 |
| mechvent_1 | 2980 | 0.93 | 1002 | 0.95 | 1978 | 0.92 | 0.001 |
| congestive_heart_failure_0 | 2306 | 0.72 | 743 | 0.70 | 1563 | 0.73 | 0.221 |
| congestive_heart_failure_1 | 900 | 0.28 | 311 | 0.30 | 589 | 0.27 | 0.221 |
| cardiac_arrhythmias_0 | 2321 | 0.72 | 724 | 0.69 | 1597 | 0.74 | 0.001 |
| cardiac_arrhythmias_1 | 885 | 0.28 | 330 | 0.31 | 555 | 0.26 | 0.001 |
| valvular_disease_0 | 2957 | 0.92 | 972 | 0.92 | 1985 | 0.92 | 1.000 |
| valvular_disease_1 | 249 | 0.08 | 82 | 0.08 | 167 | 0.08 | 1.000 |
| pulmonary_circulation_0 | 2981 | 0.93 | 975 | 0.93 | 2006 | 0.93 | 0.505 |
| pulmonary_circulation_1 | 225 | 0.07 | 79 | 0.07 | 146 | 0.07 | 0.505 |
| peripheral_vascular_0 | 2893 | 0.90 | 943 | 0.89 | 1950 | 0.91 | 0.336 |
| peripheral_vascular_1 | 313 | 0.10 | 111 | 0.11 | 202 | 0.09 | 0.336 |
| hypertension_0 | 2760 | 0.86 | 905 | 0.86 | 1855 | 0.86 | 0.839 |
| hypertension_1 | 446 | 0.14 | 149 | 0.14 | 297 | 0.14 | 0.839 |
| paralysis_0 | 3091 | 0.96 | 1031 | 0.98 | 2060 | 0.96 | 0.004 |
| paralysis_1 | 115 | 0.04 | 23 | 0.02 | 92 | 0.04 | 0.004 |
| other_neurological_0 | 2798 | 0.87 | 941 | 0.89 | 1857 | 0.86 | 0.020 |
| other_neurological_1 | 408 | 0.13 | 113 | 0.11 | 295 | 0.14 | 0.020 |
| chronic_pulmonary_0 | 2462 | 0.77 | 823 | 0.78 | 1639 | 0.76 | 0.243 |
| chronic_pulmonary_1 | 744 | 0.23 | 231 | 0.22 | 513 | 0.24 | 0.243 |
| diabetes_uncomplicated_0 | 2516 | 0.78 | 830 | 0.79 | 1686 | 0.78 | 0.830 |
| diabetes_uncomplicated_1 | 690 | 0.22 | 224 | 0.21 | 466 | 0.22 | 0.830 |
| diabetes_complicated_0 | 2972 | 0.93 | 993 | 0.94 | 1979 | 0.92 | 0.026 |
| diabetes_complicated_1 | 234 | 0.07 | 61 | 0.06 | 173 | 0.08 | 0.026 |
| hypothyroidism_0 | 2852 | 0.89 | 938 | 0.89 | 1914 | 0.89 | 1.000 |
| hypothyroidism_1 | 354 | 0.11 | 116 | 0.11 | 238 | 0.11 | 1.000 |
| renal_failure_0 | 2632 | 0.82 | 848 | 0.80 | 1784 | 0.83 | 0.100 |
| renal_failure_1 | 574 | 0.18 | 206 | 0.20 | 368 | 0.17 | 0.100 |
| liver_disease_0 | 2703 | 0.84 | 861 | 0.82 | 1842 | 0.86 | 0.005 |
| liver_disease_1 | 503 | 0.16 | 193 | 0.18 | 310 | 0.14 | 0.005 |
| peptic_ulcer_0 | 3203 | 1.00 | 1054 | 1.00 | 2149 | 1.00 | 0.550 |
| peptic_ulcer_1 | 3 | 0.00 | 0 | 0.00 | 3 | 0.00 | 0.550 |
| aids_0 | 3185 | 0.99 | 1049 | 1.00 | 2136 | 0.99 | 0.513 |
| aids_1 | 21 | 0.01 | 5 | 0.00 | 16 | 0.01 | 0.513 |
| lymphoma_0 | 3139 | 0.98 | 1017 | 0.96 | 2122 | 0.99 | 0.000 |
| lymphoma_1 | 67 | 0.02 | 37 | 0.04 | 30 | 0.01 | 0.000 |
| metastatic_cancer_0 | 3030 | 0.95 | 959 | 0.91 | 2071 | 0.96 | 0.000 |
| metastatic_cancer_1 | 176 | 0.05 | 95 | 0.09 | 81 | 0.04 | 0.000 |
| solid_tumor_0 | 3074 | 0.96 | 1005 | 0.95 | 2069 | 0.96 | 0.334 |
| solid_tumor_1 | 132 | 0.04 | 49 | 0.05 | 83 | 0.04 | 0.334 |
| rheumatoid_arthritis_0 | 3102 | 0.97 | 1018 | 0.97 | 2084 | 0.97 | 0.781 |
| rheumatoid_arthritis_1 | 104 | 0.03 | 36 | 0.03 | 68 | 0.03 | 0.781 |
| coagulopathy_0 | 2286 | 0.71 | 672 | 0.64 | 1614 | 0.75 | 0.000 |
| coagulopathy_1 | 920 | 0.29 | 382 | 0.36 | 538 | 0.25 | 0.000 |
| obesity_0 | 2977 | 0.93 | 1005 | 0.95 | 1972 | 0.92 | 0.000 |
| obesity_1 | 229 | 0.07 | 49 | 0.05 | 180 | 0.08 | 0.000 |
| weight_loss_0 | 2919 | 0.91 | 975 | 0.93 | 1944 | 0.90 | 0.050 |
| weight_loss_1 | 287 | 0.09 | 79 | 0.07 | 208 | 0.10 | 0.050 |
| fluid_electrolyte_0 | 1501 | 0.47 | 444 | 0.42 | 1057 | 0.49 | 0.000 |
| fluid_electrolyte_1 | 1705 | 0.53 | 610 | 0.58 | 1095 | 0.51 | 0.000 |
| blood_loss_anemia_0 | 3111 | 0.97 | 1031 | 0.98 | 2080 | 0.97 | 0.086 |
| blood_loss_anemia_1 | 95 | 0.03 | 23 | 0.02 | 72 | 0.03 | 0.086 |
| deficiency_anemias_0 | 2422 | 0.76 | 856 | 0.81 | 1566 | 0.73 | 0.000 |
| deficiency_anemias_1 | 784 | 0.24 | 198 | 0.19 | 586 | 0.27 | 0.000 |
| alcohol_abuse_0 | 2852 | 0.89 | 948 | 0.90 | 1904 | 0.88 | 0.236 |
| alcohol_abuse_1 | 354 | 0.11 | 106 | 0.10 | 248 | 0.12 | 0.236 |
| drug_abuse_0 | 3067 | 0.96 | 1022 | 0.97 | 2045 | 0.95 | 0.015 |
| drug_abuse_1 | 139 | 0.04 | 32 | 0.03 | 107 | 0.05 | 0.015 |
| psychoses_0 | 3051 | 0.95 | 1021 | 0.97 | 2030 | 0.94 | 0.002 |
| psychoses_1 | 155 | 0.05 | 33 | 0.03 | 122 | 0.06 | 0.002 |
| depression_0 | 2916 | 0.91 | 987 | 0.94 | 1929 | 0.90 | 0.000 |
| depression_1 | 290 | 0.09 | 67 | 0.06 | 223 | 0.10 | 0.000 |

# Table 3. The LASSO score

| Variables | -2 | 1 | 2 | 3 | 4 | 5 | 6 | 7 | 8 | 10 |
| --- | --- | --- | --- | --- | --- | --- | --- | --- | --- | --- |
| A-aO_2_ (mmHg) |  |  |  | >360 & ≤460 | >460 |  |  |  |  |  |
| Albumin (g/dl) |  |  | ≤1.8 |  |  |  |  |  |  |  |
| Bicarbonate (mmol/l) |  | ≤18 or >38 |  |  |  |  |  |  |  |  |
| Bilirubin (mg/dl) |  |  | >7 &≤10 |  | >10 |  |  |  |  |  |
| BUN (mg/dl) |  |  | >45 |  |  |  |  |  |  |  |
| Diastolic BP (mmHg) |  |  | >45 & ≤56 |  |  |  |  |  |  |  |
| Glucose (mg/dl) |  |  | ≤80 |  |  |  |  |  |  |  |
| Heart rate (per minute) |  | >90 & ≤130 |  |  |  |  |  |  |  |  |
| INR |  |  |  | >1.8 |  |  |  |  |  |  |
| Lactate (mmol/l) |  | >2.2 & ≤4.7 |  | >6.2 & ≤7.9 |  |  |  |  |  | >7.9 |
| PaCO_2_ (mmHg) |  | >47 |  |  |  |  |  |  |  |  |
| pH |  | ≤7.26 |  |  |  |  |  |  |  |  |
| Platelet ($\boldsymbol{\times}\boldsymbol{10}^{\boldsymbol{9}}\boldsymbol{/}\mathbf{l}$) |  | >110 &≤150 | >70 & ≤110 |  |  |  |  |  | ≤70 |  |
| PaO_2_ (mmHg) |  |  |  |  | ≤70 |  |  |  |  |  |
| aPTT (s) |  |  | >42.5 |  |  |  |  |  |  |  |
| Respiratory rate (per minute) |  | >19 & ≤26 |  | >26 & ≤41.5 |  |  |  |  |  |  |
| Sodium (mmol/l) |  |  | ≤127 |  |  |  |  |  |  |  |
| Systolic BP (mmHg) |  | >105 & ≤115 |  | >90 & ≤105 |  |  |  | ≤90 |  |  |
| Temperature (℃) |  | >35.7 & ≤36 |  |  |  | ≤35.7 |  |  |  |  |
| WBC ($\boldsymbol{\times}\boldsymbol{10}^{\boldsymbol{9}}\boldsymbol{/}\mathbf{l}$) |  |  |  | ≤2 |  |  |  |  |  |  |
| Age (years) |  |  |  | >55 & ≤80 |  |  |  | >80 |  |  |
| Minimum GCS |  |  |  | ≤5 |  |  |  |  |  |  |
| Urine output (ml/24h) |  |  | >400 & ≤ 1200 |  | >100 & ≤400 |  | ≤100 |  |  |  |
| Dopamine (mg/kg/min) |  |  |  | >10 |  |  |  |  |  |  |
| Epinephrine (mg/kg/min) |  | >0.12 |  |  |  |  |  |  |  |  |
| Norepinephrine (mg/kg/min) |  |  |  |  | >0.3 |  |  |  |  |  |
| Emergency admission |  |  | Yes |  |  |  |  |  |  |  |
| Mechanical ventilation |  |  |  |  | Yes |  |  |  |  |  |
| Cardiac arrhythmias |  | Yes |  |  |  |  |  |  |  |  |
| Lymphoma |  |  |  |  |  |  | Yes |  |  |  |
| Metastatic cancer |  |  |  |  |  |  |  | Yes |  |  |
| Obesity | Yes |  |  |  |  |  |  |  |  |  |

Abbreviations: A-aO_2_: alveolar–arterial gradient; BUN: blood urea nitrogen; BP: blood pressure; INR: international normalized ratio; PaCO_2_: partial pressure of arterial carbon dioxide; PaO_2_: partial pressure of arterial oxygen; GCS: Glasgow Coma scale; WBC: white blood cell; aPTT: activated partial thrombin time.
